# Nanostructured Catalysts for Electro- and Photocatalytic Energy Conversion: Design Strategies, Mechanistic Descriptors, and Practical Applications

**DOI:** 10.3390/nano16130788

**Published:** 2026-06-23

**Authors:** Xiangjun Kong, Xia Wang, Wulan Zeng

**Affiliations:** School of Chemistry & Chemical Engineering and Environmental Engineering, Weifang University, Weifang 261061, China

**Keywords:** nanostructured catalysts, electrocatalysis, photocatalysis, water splitting, CO_2_ reduction, fuel cells, metal–air batteries

## Abstract

Nanostructured catalysts have become a core component of energy conversion in electrocatalysis and photocatalysis; however, successfully translating their performance from laboratory scale to industrial applications remains a long-standing challenge. This paper provides a critical assessment of the field, systematically tracing the entire development trajectory from catalyst design to practical application. We focus on five major classes of catalysts—monometallic catalysts, bimetallic/multimetallic alloy catalysts, metal compound catalysts, carbon-based composite catalysts, and single-atom catalysts—and explore synthetic strategies for achieving precise structural control, including hydrothermal/solvothermal methods, electrodeposition, template-assisted and MOF-derived syntheses, high-temperature pyrolysis, and post-treatment defect engineering. This paper delves into the mechanisms and performance descriptors governing the hydrogen evolution reaction (HER), oxygen evolution reaction (OER), oxygen reduction reaction (ORR), urea oxidation, photocatalytic water splitting, and CO_2_ reduction. Based on the above analysis, this paper lays the mechanistic foundation for five core strategies to improve catalyst performance: morphology control, elemental doping, heterostructure and interface engineering, defect and vacancy engineering, and support modification. Furthermore, this paper provides an in-depth evaluation of the applications of these catalysts in water splitting, CO_2_ valorization, fuel cells, metal–air batteries, and energy-saving electrolysis, with a particular focus on earth-abundant alternatives to precious metals. We argue that in many well-studied reactions, intrinsic activity may no longer be the primary bottleneck restricting their development; instead, the core challenge now lies in maintaining excellent catalytic performance under harsh and industrially relevant conditions, especially under high-current densities, impurity-containing feed systems, and long-term operating conditions. In response to this shift in research focus, this paper clearly identifies the key obstacles hindering the industrial application of catalysts and proposes practical directions for future research.

## 1. Introduction

The transition from fossil fuels to renewable energy is one of the most important scientific and social challenges of this century [1,2]. Electrocatalysis and photocatalysis are key technologies for sustainable energy conversion, and semiconductor photocatalysis has also been extended to related light-driven chemical transformations [3]. These technologies provide a viable pathway for storing intermittent solar and wind energy under mild conditions and with the potential for low-carbon operation, particularly in the form of chemically bonded products such as hydrogen and reduced carbon products [4,5].

The performance of these systems is heavily dependent on the catalyst. The catalyst determines the overpotential, controls the branching pathways of the products, and ultimately determines how much of the input energy is converted into the desired fuel [6]. Over the past decade, this field has shifted from bulk metals and oxides to nanostructured systems. At the nanoscale, materials can achieve high specific surface areas, increase the density of undercoordinated sites, and possess electronic structures that can be tuned by size, shape, and interfacial strain. These features can contribute to improved charge-transfer efficiency and optimized reaction rates [7,8,9].

The progress is evident. In oxygen evolution (OER), hydrogen evolution (HER), and selected CO_2_ electrolysis systems, earth-abundant nanostructured catalysts have achieved performance comparable to selected precious metal benchmarks under optimized laboratory conditions. This was first demonstrated for OER [10] and soon after, for HER and CO_2_ reduction. In photocatalysis, significant improvements in product yield and selectivity have been achieved in decoupled light–dark hydrogen production [11] and CO_2_-to-fuel conversion [12]. Meanwhile, the application of operando characterization techniques and theoretical models is beginning to reveal the mechanistic basis for these performance improvements, bringing this field closer to true rational design.

Twenty years of research have led these catalysts to a stage where further improvements in catalytic activity may no longer be the only primary goal in several well-studied reactions. The more pressing challenge now is maintaining existing performance under the conditions required for practical applications, such as industrial current densities, impurity-containing feedstock flows, and extended continuous operation. This review adopts this perspective. Despite many excellent review papers that have been written on nanostructured catalysts, single-atom catalysts, transition-metal compounds, MOF-derived catalysts, photocatalytic CO_2_ reduction, oxygen reduction reaction, water splitting, and industrially relevant electrocatalysis, the majority of the papers revolve around a particular catalyst category, catalyst synthesis method, reaction type, or application. Hence, the relationship between catalyst classification, structure evolution controlled by catalyst synthesis, reaction-specific descriptors, performance improvement mechanisms, benchmarking, and translation challenges at the application level tends to be discussed in isolation. This review differs from the existing literature by bringing together the concepts of electro- and photocatalytic energy conversion through a unified design-to-application perspective. Instead of providing a comprehensive review of individual catalyst systems, the current review highlights the role of nanoscale structure control in active-site exposure, electronic configuration, interfacial charge transfer, intermediate adsorption, product selectivity, and stability for various reactions. The transition from activity-driven catalyst development to application-oriented assessment is given particular importance. In this connection, durability, real operating conditions, catalyst deployment, scale-up possibilities, as well as suitability for devices, cost, and life-cycle effects are among the factors considered in assessing practical catalyst effectiveness. The positioning of the present review relative to representative previous reviews is summarized in Table 1.

Accordingly, this review outlines the main catalyst families (Section 2) and synthesis methods for achieving nanostructure control (Section 3), discusses reaction mechanisms and performance descriptors that link structure and performance (Section 4), and evaluates five major enhancement strategies based on the aforementioned mechanisms (morphology control, elemental doping, heterostructure and interface engineering, defect and vacancy engineering, and support modification) (Section 5). This review also discusses the applications of catalysts in water splitting, CO_2_ valorization, fuel cells, metal–air batteries, and energy-saving electrolysis, focusing on earth-abundant alternatives to precious metals (Section 6). Section 7 systematically details the practical challenges and future development prospects in the process from laboratory success to industrial application.

**Figure 1:**
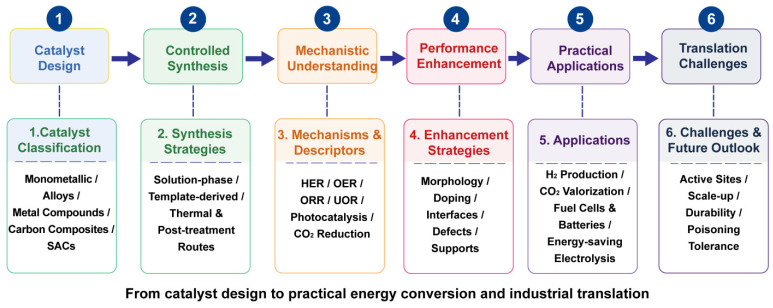
Overall framework of the review: from catalyst design to industrially relevant energy conversion. The review is organized from catalyst design and controlled synthesis to mechanistic understanding, performance enhancement, practical applications, and translation challenges.

To provide a clear roadmap for the discussion, the overall organization of this review is summarized in Figure 1. The review proceeds from catalyst classification and synthesis strategies to reaction mechanisms, performance enhancement approaches, representative applications, and finally, the practical challenges associated with industrial translation.

## 2. Classification of Nanostructured Catalysts in Energy Conversion

**Figure 2:**
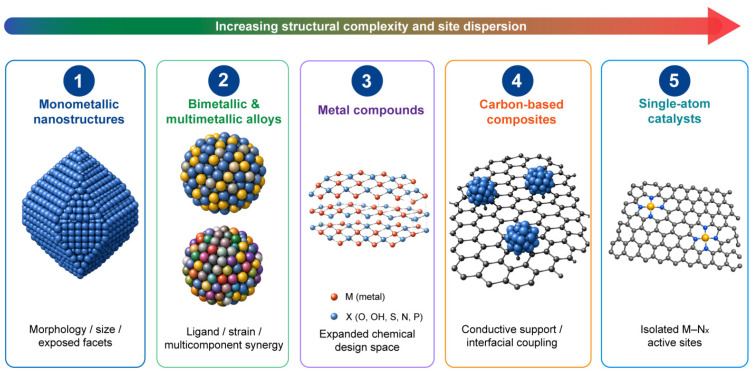
Classification of nanostructured catalysts for energy-conversion reactions. The five catalyst families are arranged according to increasing structural complexity and site dispersion. In the bimetallic and multimetallic alloy panel, differently colored spheres schematically denote different metallic elements.

In this article, nanostructured catalysts are classified into five types. Ordered from simpler to more complex structures, these categories include monometallic systems, in which activity depends mainly on morphology and an exposed surface; bimetallic and multimetallic alloys, where there are synergistic effects associated with the modification of electronic properties; metal compounds that significantly extend the catalyst design space; and carbon-based composite materials and single-atom catalysts, in which active-site dispersion approaches the limit. These classifications overlap substantially since the highest activity is observed when multiple principles are utilized simultaneously. However, this classification still allows us to discuss common features of the mentioned categories. This classification framework is schematically summarized in Figure 2, which illustrates the representative structural motifs and site-dispersion features of the five catalyst families.

### 2.1. Transition-Metal-Based Monometallic Catalysts

The simplest monometallic catalysts are those consisting of 3d transition metals (Ni, Co, Fe, Cu, and Mo). These are among the first electrocatalysts tested for energy-conversion applications. One of the early achievements was the in situ generation of cobalt-based oxygen evolution catalysts using phosphate-buffered neutral water, demonstrating that non-noble elements can reach performance comparable to noble-metal benchmarks in selected systems [33]. Recent applications are also promising. For example, nickel-catalyzed hydrogenation of CO_2_ to methanol in conditions compatible with industry was recently demonstrated [34]. While platinum-group catalysts often show high intrinsic activity, transition-metal catalysts offer advantages in cost and scalability. Thus, they are expected to compete effectively with noble-metal catalysts [35]. Morphology, exposed surface, and defect structure of the catalyst are essential parameters influencing its activity. In particular, nanostructures based on nickel demonstrate excellent activity in hydrogen and oxygen evolution reactions (HER, OER) [36].

### 2.2. Bimetallic/Multimetallic Alloy Catalysts

Bimetallic and multimetallic alloy catalysts include another component besides the active metal in their lattice. Electronic interactions and geometric effects contribute to the modulation of the binding energy of intermediates [37]. The main principle has already been elaborated. Shifts in the d-band position can change the binding strength of reaction intermediates, and in some cases, help to alleviate the linear scaling relationships that limit monometallic catalysts. Current benchmarks of non-noble-metal oxygen evolution reaction (OER) catalysts and water-splitting catalysts include NiFe, NiCo, and CoFe alloys [16]. Bimetallic catalysts may show enhanced catalytic activity in other processes as well. Thus, Liu et al. demonstrated that bimetallic AuRu nanoparticles immobilized on ZIF-67 are highly efficient in alcohol oxidation due to the strong electronic interaction between these elements [38]. Extension of this principle to multimetallic systems gives rise to a broad set of options for tuning catalytic performance through control of the electronic structure of catalysts. Zhang et al. proposed a multilayer porous high-entropy alloy NiFeCoZn catalyst based on an fcc alloy structure [39]. The synergistic interaction between Ni, Fe, Co, and Zn elements due to lattice strain and high-entropy stabilization results in high bifunctional HER/OER catalytic activity. Moreover, this catalyst is capable of splitting water under a cell voltage of 1.51 V. High stability for over 1000 h further supports potential industrial applications. Research on multimetallic and high-entropy alloy catalysts is a promising future trend [17].

### 2.3. Metal Compound Catalysts

Metal compounds (oxides, hydroxides, sulfides, phosphides, nitrides, and carbides) represent one of the most widely studied classes of non-noble energy-conversion catalysts [40]. Their wide applicability is associated with rich electronic states and, in selected cases, improved stability in different electrolyte environments. NiFe-layered double hydroxides (LDHs) have been extensively used in oxygen evolution reactions due to their easy synthesis, low price, and high intrinsic activity [18]. Another popular class of metal compound catalysts includes cuprite Cu_2_O-based composite materials with photocatalytic activity [41]. Hydrogen evolution reactions (HERs) and water splitting are facilitated by sulfides and phosphides. Aside from their excellent conductivity, their metallic state provides good hydrogen adsorption energy [23]. Nitrides and carbides have both of these properties. High electrical conductivity and corrosion resistance are especially important for industrial-scale electrolysis in high-current densities [42].

### 2.4. Carbon-Based Composite Catalysts

This category includes conductive carbon matrix (graphene, carbon nanotube, porous carbon, or carbon nitride) combined with a metal-derived active phase [43]. A carbon-based matrix provides a high surface area for dispersion of catalytically active metal particles, accelerates electron transfer, and prevents nanoparticle aggregation, improving stability and activity [44]. Graphene and graphitic carbon nitride (g-C_3_N_4_) are among the most frequently used substrates. Metal nanoparticles supported by graphene are widely used in electrolyzers and fuel cells. On the other hand, g-C_3_N_4_, a metal-free polymeric semiconductor, is remarkable in photocatalysis. Due to its suitable band gap and chemical stability, it is one of the most widely studied carbon-based platforms for visible-light photocatalysis and can serve both as a support material for co-catalysts and as an effective light absorber. The versatility of this material also manifests itself in homojunctions. In case of type II junctions, pure carbon nitride combined with cyano-rich boron-doped carbon nitride allows achieving bifunctionality, including hydrogen and selectively oxidizing benzyl alcohol [45].

Despite their abundance, tunable conductivity, and structural versatility, carbon-based electrocatalysts still face several challenges before practical application. At the laboratory scale, the real active sites in doped carbon, metal–carbon composites, and M–N–C structures are often difficult to identify unambiguously because activity may arise from heteroatom dopants, defects, metal nanoparticles, atomically dispersed metal sites, or their coupled interfaces. At the large-scale level, additional concerns include carbon corrosion under oxidative potentials, loss or restructuring of active sites, instability of heteroatom dopants, pore-structure collapse, batch-to-batch variations, and the difficulty of integrating powder catalysts into robust large-area electrodes. Future research on carbon-based electrocatalysts should therefore combine active-site identification, corrosion-resistant carbon architectures, controlled doping, scalable synthesis, and electrode-level evaluation rather than focusing only on apparent activity in small-scale tests.

### 2.5. Single-Atom and Dual-Atom Catalysts

Single-atom catalysts (SACs) can achieve a theoretical atom utilization efficiency of 100% by isolating active metal sites on the surface of a support [46]. In comparison with traditional nanoparticles, SACs allow not only for maximizing the exposure of the catalytically active site, but they also serve as a platform for studying the structure–activity relationship [47]. Significant progress has been made in the development of SACs in the fields of electrochemical energy conversion and photocatalysis. Platinum (Pt)-, iridium (Ir)-, and ruthenium (Ru)-based catalysts achieve comparable performance to commercial nanoparticles with a dramatically reduced amount of metal on the substrate. Other non-noble-metal single atoms (e.g., Fe, Co, Ni, or Cu) stabilized on nitrogen-doped carbon have demonstrated promising oxygen reduction reaction (ORR), CO_2_ reduction, and hydrogen evolution reaction (HER) activity [13,14,48,49]. Recently, dual-atom catalysts, which bind adjacent metal centers, have been introduced as well. Synergistic catalytic activity at dual-atom sites may help alleviate scaling-relation constraints and potentially improve performance beyond isolated single-atom catalysts [15,50]. The key structural features, design variables, and major design concerns of the five catalyst families discussed in this section are summarized in Table 2.

Overall, the catalyst families summarized above should not be regarded as a simple hierarchy in which structural complexity automatically leads to higher practical value. Monometallic catalysts provide relatively simple platforms for clarifying structure–activity relationships, but their electronic tunability is limited. Multimetallic alloys and metal compounds offer broader opportunities for adsorption-energy regulation and redox-state modulation, yet they often undergo surface segregation, phase reconstruction, or dynamic transformation under operation. Carbon-based composites and single-atom/dual-atom catalysts maximize dispersion and interfacial contact, but they introduce additional uncertainties related to active-site identification, metal–support interactions, carbon corrosion, and site stability. Therefore, the rational selection of a catalyst family should depend not only on intrinsic activity but also on the reaction environment, durability requirements, structural traceability, and scalability.

## 3. Common Synthesis Strategies for High-Performance Nanostructured Catalysts

**Figure 3:**
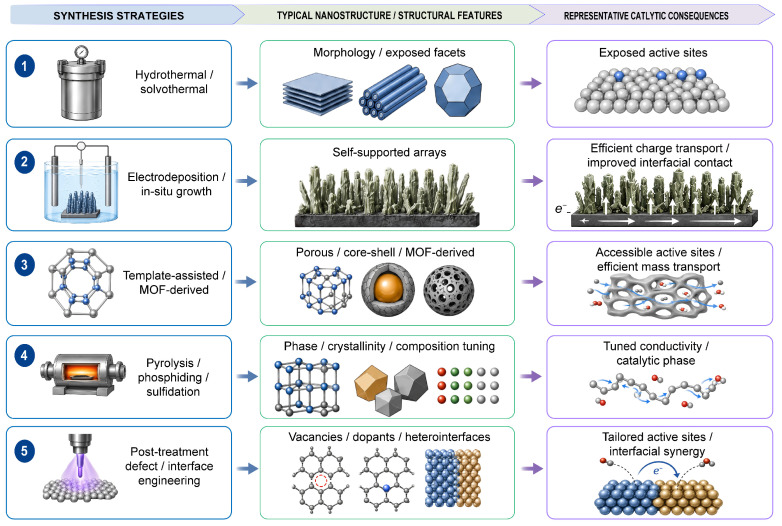
Synthesis–structure–property relationship of common synthesis strategies for nanostructured catalysts. The figure summarizes five representative synthesis routes and their associated structural features and catalytic consequences. Synthetic routes are often combined in practice, and scalability should be considered alongside structural precision. Different colored spheres schematically represent different atomic species, and red dashed circles indicate representative defect or vacancy sites.

The physicochemical properties responsible for catalytic performance (exposed crystal facets, defect density, electronic structure, interface structure, etc.) are significantly influenced by the synthesis procedure. Regarding nanocatalysts for electrocatalysis and photocatalysis, five synthesis procedures stand out regarding versatility. They can be loosely organized by the environment in which nanostructure formation occurs: solution-phase methods (hydrothermal/solvothermal synthesis and electrodeposition/in situ growth), templating approaches (template-assisted and MOF-derived routes), and post-synthetic treatments that introduce functionality through thermal or chemical processing (high-temperature pyrolysis, phosphiding/sulfidation, and defect engineering) [19]. However, it should be noted that the lines between them are more blurred than this classification suggests: MOF-derived catalysts usually require post-synthesis thermal decomposition, and products from hydrothermal synthesis need post-synthesis defect engineering [20]. This section examines each strategy, with an emphasis placed on how synthetic parameters translate into structural features and, ultimately, into catalytic function. This synthesis–structure–property relationship is schematically summarized in Figure 3, providing a visual framework for the synthesis strategies discussed below.

### 3.1. Hydrothermal and Solvothermal Syntheses

Hydrothermal and solvothermal syntheses are the most common solution-phase synthesis methods for producing catalysts. Specifically, this process usually involves placing a liquid precursor (aqueous solution or organic solution) into an autoclave and heating it to 100–220 °C. This heating initiates autogenic pressure and contributes to nucleation and crystal growth [51]. As already mentioned, the primary advantage of these methods lies in great flexibility: the properties of the solvent, concentration and composition of the precursor, temperature, reaction time, and the use of capping agents enable the fine-tuning of morphology, crystal planes, and the sizes of catalysts. In particular, the solvent determines the thermodynamic driving force of nucleation and crystal growth rate due to its polarity, viscosity, and coordination ability, making this method superior to other solution-phase syntheses [52]. The only problem associated with hydrothermal methods is autogenic pressure and the inability to significantly scale up the reactor. In addition, the homogeneity of products is a difficult task to accomplish in mass production (Section 7) [53].

This approach applies to nearly all catalyst types discussed in Section 2: transition metal nanosheets and nanoparticles, hollow structures, and hierarchical composite materials. Typical examples include NiFe-layered double hydroxide nanosheets, Co_3_O_4_ nanoparticles, and many Mn-based bimetallic complexes [54,55]. The conditions under which hydrothermal synthesis occurs contribute to the formation of ultrathin structures and the exposure of an enormous number of active sites [56]. Ideally, heterostructure catalysts can be produced via a one-pot hydrothermal synthesis step [57].

### 3.2. Electrodeposition and In Situ Growth

The method of electrodeposition can be considered an efficient, easy-to-scale-up, and inexpensive way to produce self-supporting electrocatalysts. Recently, it was even proven to be successful in producing high-entropy alloys [58]. Essentially, the method of electrodeposition consists of passing a constant potential or current through the solution with ions of a catalytically active metal, reducing it, and depositing a dense or porous thin layer of nanostructured metal on the surface of a conductive substrate (usually nickel foam, carbon cloth, or copper foil).

Similarly, in situ growth methods, that is, hydrothermal anchoring or electrochemical deposition, help prevent nanoparticle aggregation and improve structural integrity. It has already been shown that in situ methods can be used successfully for preparing catalysts for the OER and HER, such as Cu_3_N nanorod arrays grown on copper foam [59]. The thickness, loading, and roughness of catalyst coatings can be controlled by varying the deposition time and charge density [60]. Dealloying is another in situ technique that can lead to the formation of hierarchical porous metal foams through selective dissolution of alloy precursors [61].

### 3.3. Template-Assisted Synthesis and MOF Derivation

The technique of template-assisted synthesis is suitable for the preparation of hollow and regularly porous nanostructures, as well as the core/shell structures of the material, due to its high flexibility. In particular, transition-metal phosphide electrocatalysts usually demonstrate multifunctionality within template-assisted synthesis [62]. The rigid templates (e.g., SiO_2_ spheres or polystyrene microspheres) and soft ones (e.g., surfactants and block copolymers) provide confined space for nucleation and subsequent growth, which is removed by calcination or chemical etching [63,64].

Recently, MOF derivation has gained widespread popularity due to the extraordinary activity of catalysts produced by this method. For instance, Meng et al. synthesized highly porous and highly efficient OER-active cobalt metal–organic frameworks [65]. Such materials have several advantages, including ultra-high porosity, tunability of metal sites and heteroatoms, and high uniformity of heteroatoms. Finally, after high-temperature pyrolysis, they result in defect-rich and highly conductive carbon-coated metal nanoparticles or oxides, sulfides, and phosphides. For instance, Lu et al. embedded Co nanoparticles in N-doped porous carbon from ZIF-67 through MOF-mediated pyrolysis and achieved excellent oxygen evolution reaction (OER) catalytic activity [66]. Wu et al. also prepared Co-nanoparticle-embedded carbon by a one-step pyrolysis method for effective water oxidation [67]. In addition to improving structural stability, this method can prevent leaching of active metals during long-term operation and thus ensure better industrial applicability [68].

### 3.4. High-Temperature Pyrolysis, Phosphiding, and Sulfidation

Another popular synthesis approach is high-temperature pyrolysis (sometimes coupled with phosphiding or sulfidation processes). This method can produce very conductive and crystalline transition-metal catalysts, which are a powerful complement to MOF-based catalysts [69]. It involves high-temperature processing of the precursor in an inert atmosphere of N_2_ or Ar. In this case, the carbonization of the ligand and the subsequent carbothermal reduction of metal ions lead to the formation of the desired crystal phase. At this stage, phosphorous or sulfur atoms are introduced to regulate the electronic state of the active metal sites and optimize the binding energy of the intermediates of OER and HER, increasing catalytic activity dramatically [70].

Many high-performing non-noble-metal electrocatalysts can be prepared through this approach. For instance, typical transition-metal phosphides and sulfides (CoP, Ni_2_P, FeS_2_, Ni_3_S_2_) showed much higher bifunctional catalytic activity in OER and HER after phosphiding or sulfidation [71,72,73,74,75]. Additionally, high-temperature heat treatment can improve the crystallinity of materials and decrease grain boundary scattering, which increases conductivity and accelerates interfacial charge transfer [76].

### 3.5. Defect and Interface Engineering via Post-Treatment

Post-treatment methods such as plasma etching, chemical reduction, thermal annealing, and acid/base etching are quite popular when producing oxygen-vacancy-rich oxides, nitrogen-doped carbon composites, and modified heterostructures [77,78]. Through defect engineering, the catalytic d-band center can be shifted to facilitate charge separation and increase active sites [79,80,81].

Typical results of post-treatment approaches include oxygen vacancy-containing oxide materials, nitrogen-doped carbon composites, and modified heterostructures [82]. For example, defect engineering significantly improved the catalytic activity of oxygen-vacancy-rich Co_3_O_4_ nanocomposites prepared via hydrothermal synthesis [83], while the oxygen vacancies increased the catalytic activity of dendritic structure Co(OH)_2_ for the oxygen evolution reaction [84]. Moreover, defect engineering strategies are highly compatible with the pre-prepared nanostructures, forming a powerful universal tool that increases the activity of a material [85].

None of the presented methods is equally effective in satisfying all requirements. While hydrothermal synthesis shows high flexibility in controlling morphology, scaling up remains difficult; moreover, electrodeposition requires conductive substrates but produces strongly bonded electrodes and catalysts. Metal–organic framework-derived catalysts allow the formation of high-surface-area structures, but careful control of pyrolysis conditions is necessary to preserve their structure. Defect engineering through post-treatment can enhance catalytic performance by introducing defects; however, the resulting metastable structures may affect material stability. The major synthesis strategies, as well as their structural outcomes, advantages, and practical concerns, are summarized in Table 3. Ultimately, the choice of synthetic method is essential, as it defines the nanostructures, electronic states, and catalyst performance explored in future research. Importantly, the scalability of the synthetic process should not be treated as a late-stage engineering issue, but it should be considered from the earliest stages of catalyst development, together with structural precision, as discussed further in Section 7.

A critical comparison of these synthesis strategies indicates that structural precision and practical scalability are often in tension. Hydrothermal and solvothermal methods provide flexible morphology control but are limited by batch-to-batch variation and pressure-dependent scale-up. Electrodeposition and in situ growth are more compatible with electrode fabrication, yet they are strongly substrate-dependent and require careful control of film uniformity over large areas. MOF-derived, template-assisted, and high-temperature conversion routes can produce highly porous or compositionally uniform architectures, but their multistep processing, template removal, pyrolysis conditions, and possible pore collapse may increase manufacturing complexity. Post-treatment defect engineering is especially powerful for activity optimization, but the resulting defect-rich structures can be metastable during long-term operation. Thus, the most suitable synthesis strategy is not necessarily the one that gives the highest activity in a small-scale test, but the one that balances structural control, reproducibility, durability, cost, and compatibility with device fabrication.

## 4. Classic Catalytic Mechanisms and Key Performance Descriptors

As mentioned in Section 3, the synthetic pathway defines the geometry, electronic state, and interfacial properties of nanostructured catalysts. It then influences the behavior of the material in energy-conversion reactions [86]. The development from an experience-driven trial-and-error approach to a knowledge-oriented rationale relies heavily on a profound understanding of the mechanisms and the quantifiable descriptors that link the structure and performance of the materials [87]. Whether electrocatalysis or photocatalysis, the processes include mass transport, reactant adsorption, charge transport, intermediate conversion, product desorption, and sometimes even surface reconstruction, forming the basis of descriptor-based optimization strategies [88]. This section presents the classic reaction mechanisms of representative electrocatalysis and photocatalysis, introduces essential performance descriptors, and highlights their role as the theoretical foundation for the enhancement strategies discussed in Section 5. Despite differences in the driving forces (bias voltage in the case of electrocatalysis, photogenerated charge carriers for photocatalysis), similar criteria for surface chemistry govern the activity and selectivity of the reactions, such as the adsorption energy of intermediates, charge transport efficiency at the catalyst–electrolyte interfaces, and surface stability of active sites under practical operation. The realization of such commonality is essential since it enables the application of mechanistic knowledge gained from one type of catalytic reaction to another, which shall be fully exploited in the discussion of enhancement strategies in Section 5.

### 4.1. Mechanisms and Core Descriptors for Electrocatalysis

**Figure 4:**
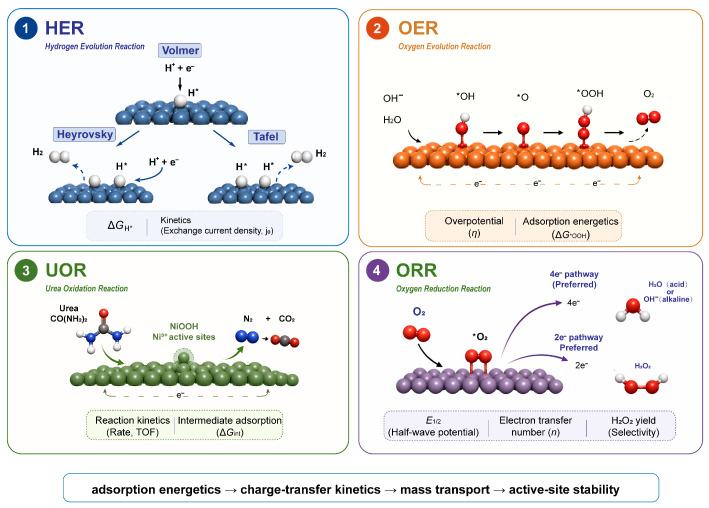
Core electrocatalytic mechanisms and representative descriptors of HER, OER, UOR, and ORR. Representative reaction pathways, key intermediates, and commonly used performance descriptors are summarized for the hydrogen evolution reaction (HER), oxygen evolution reaction (OER), urea oxidation reaction (UOR), and oxygen reduction reaction (ORR). The bottom schematic highlights shared catalytic factors across these reactions, including adsorption energetics, charge-transfer kinetics, mass transport, and active-site stability. Different colored spheres schematically represent different atomic species involved in the reaction intermediates.

Electrocatalysis takes place at the triple phase boundary of the catalyst/electrolyte/conductive substrate. The efficiency and kinetics of the whole process largely depend on the rate of charge transfer, the equilibrium adsorption/desorption of intermediates, and the mass transfer capability of the catalyst [89]. Among various reactions for clean energy conversion, prominent candidates include the hydrogen evolution reaction (HER), oxygen evolution reaction (OER), oxygen reduction reaction (ORR), and urea oxidation reaction (UOR) as alternative energy-efficient processes of oxygen evolution. The principal electrocatalytic reactions discussed in this review are schematically summarized in Figure 4, which highlights their key reaction pathways and representative descriptors.

#### 4.1.1. Hydrogen Evolution Reaction (HER)

The hydrogen evolution reaction (HER) serves as the cathode reaction in water splitting and is the simplest two-electron transfer reaction. The reaction mechanism is well established [90]. The reaction pathway for HER in aqueous solution involves either the Volmer–Heyrovsky or Volmer-Tafel pathway and mainly consists of the following three basic steps:

The Volmer step (electrochemical adsorption):

Acidic: H_3_O^+^ + * + e^−^ → H* + H_2_O (1)

Alkaline: H_2_O + * + e^−^ → H* + OH^−^ (2)

The Heyrovský step (electrochemical desorption):

Acidic: H* + H_3_O^+^ + e^−^ → H_2_ + H_2_O + * (3)

Alkaline: H* + H_2_O + e^−^ → H_2_ + OH^−^ + * (4)

The Tafel step (chemical desorption):

2H* → H2 + 2*(5)

The rate-determining step is determined by the hydrogen adsorption free energy (Δ*G*_H*_) on the surface of the catalyst—the sole descriptor that determines the activity for HER [91]. For catalysts without noble metals that are inherently less conductive, electron accessibility at the catalyst–electrolyte interface becomes equally or even more important than Δ*G*_H*_ as the dominant parameter in controlling the rate [92].

#### 4.1.2. Oxygen Evolution Reaction (OER) and Urea Oxidation Reaction (UOR)

The oxygen evolution reaction (OER) represents the anodic half-reaction of water splitting. As a four-electron, four-proton process, it is intrinsically sluggish, making the high overpotential the principal source of inefficiency for water splitting [93]. Under alkaline conditions, the adsorbate evolution mechanism (AEM) takes place, involving four sequential proton-coupled electron transfer steps (where * denotes a surface-active site):

* + OH^−^ → *OH + e^−^ (6)

*OH + OH^−^ → *O + H_2_O + e^−^ (7)

*O + OH^−^ → *OOH + e^−^ (8)

*OOH + OH^−^ → * + O_2_ + H_2_O + e^−^ (9)

The scaling relationship between the adsorption energies of *OOH and *OH intermediates limits the minimal theoretical overpotential. Thus, the difference between the adsorption energy of *O and *OH species (ΔG_*O_ − ΔG_*OH_) becomes a commonly employed descriptor for OER activity [94]. Among transition-metal-based materials, NiFe-layered double hydroxides (LDHs) are regarded as the benchmark for non-noble-metal oxygen evolution due to their optimized intermediate adsorption energy via synergistic electronic interactions—an idea which has been well demonstrated in recent composite material designs [95]. By in situ growth followed by sulfidation to prepare (NiFe)S_2_/graphene composites, Ren et al. modulated the adsorption energy of the intermediates with the synergistic effect of the two metals and the conductive graphene matrix, realizing outstanding OER performance [96].

Compared with the oxygen evolution reaction (OER), urea oxidation (UOR) is more energy-efficient with a lower theoretical equilibrium potential (0.37 V vs. RHE) than the OER (1.23 V vs. RHE), making UOR an appealing candidate for reducing the cell voltage for hydrogen production [97]. UOR requires a six-electron transfer and rupture of the C-N bond; hence, it has more complicated kinetics compared with OER. Once again, NiFe-based catalysts show great activity with the Ni^3+^ site considered as the active center for urea dehydrogenation. Yu et al. constructed a CoSe@NiFe heterostructure and investigated the synergistic effects from the two elements in urea dehydrogenation and oxidation reactions [98].

#### 4.1.3. Oxygen Reduction Reaction (ORR)

The ORR is a pivotal cathode reaction in fuel cells and metal–air batteries, which directly affects the energy efficiency and longevity of the devices [27]. Under alkaline conditions, the ORR has two possible pathways, i.e., the favorable four-electron pathway (O_2_ + 2H_2_O + 4e^−^ → 4OH^−^) or the two-electron pathway producing peroxide intermediate ions (O_2_ + H_2_O + 2e^−^ → HO_2_^−^ + OH^−^) [28]. Key performance descriptors include half-wave potential (*E*_1_/_2_), limiting current density, and *OH adsorption free energy (ΔG_*OH_), with the latter determining the selectivity of the reaction pathway and intrinsic activity—as demonstrated in recent studies of p-block single-atom ORR catalysts [99].

It should be noted that the two-electron ORR pathway is not only an undesired peroxide-generating side reaction in fuel cells, but it can also be deliberately exploited for decentralized H_2_O_2_ production. H_2_O_2_ is an important green oxidant and chemical intermediate, with applications in environmental remediation, disinfection, pulp and textile processing, and fine chemical synthesis. From a catalyst design perspective, selective 2e^−^ ORR requires stabilization of the *OOH intermediate while avoiding excessive O–O bond cleavage and a further reduction to H_2_O. Therefore, the same ORR pathway can have different design targets depending on the application: fuel-cell and metal–air battery cathodes generally require suppression of peroxide formation and promotion of the 4e^−^ pathway, whereas H_2_O_2_ electrosynthesis requires high 2e^−^ selectivity, high Faradaic efficiency, and stable operation. In addition to the electrocatalytic routes, photocatalytic and photoelectrocatalytic systems can also generate H_2_O_2_ through O_2_ reduction, driven by photogenerated electrons, providing a solar-assisted pathway for peroxide production. This distinction further highlights the importance of evaluating ORR catalysts, not only by activity metrics such as *E*_1/2_, but also by the electron-transfer number, peroxide yield, Faradaic efficiency, and product selectivity [100].

### 4.2. Photocatalytic Reaction Mechanism and Core Descriptors

**Figure 5:**
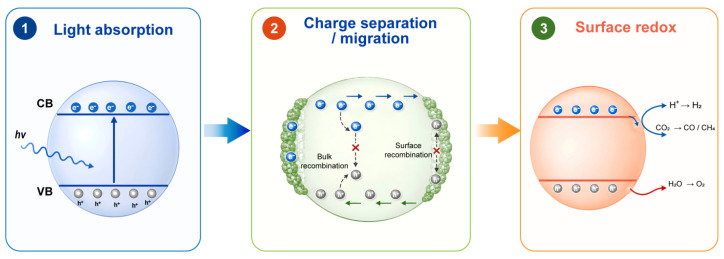
Photocatalytic mechanisms involving light absorption, charge-carrier separation/migration, and surface redox reactions. The three panels illustrate photon absorption and electron–hole generation, charge-carrier separation/migration with possible recombination losses, and representative surface reduction and oxidation reactions driven by photogenerated electrons and holes.

It can be divided into three sequential steps, which affect the efficiency of the overall process: (1) photo-absorption by semiconductor materials to create electron–hole pairs; (2) separation and transportation of photogenerated carriers to the surface; and (3) redox reactions conducted at active centers of the surface, as schematically illustrated in Figure 5 and elaborated in recent reviews on metal-based photocatalysts [101].

Photocatalysis begins with photon absorption by a semiconductor whose band gap matches the incident light energy. When the photon energy exceeds the band gap, electrons in the valence band (VB) are excited to the conduction band (CB), leaving holes in the VB and generating electron–hole pairs. This initial step determines the upper limit of solar utilization and is governed by the optical absorption coefficient, band gap, density of defect states, and the energetic positions of the CB and VB. For water splitting and CO_2_ reduction, the CB edge must be sufficiently negative to drive proton or CO_2_ reduction, whereas the VB edge must be sufficiently positive to oxidize water, hydroxide ions, or sacrificial donors. Therefore, an ideal photocatalyst should simultaneously possess broad visible-light absorption, appropriate band-edge potentials, efficient carrier separation, and chemically stable active surface sites.

Nanostructuring provides several routes to improve this first stage of photocatalysis [24,102,103]. Reducing the particle size or constructing ultrathin nanosheets shortens the diffusion distance of photogenerated carriers from the bulk to the surface, thereby decreasing the probability of bulk recombination. Porous and hollow architectures enhance light harvesting through multiple scattering and increase the density of accessible surface sites. Elemental doping and defect engineering can introduce mid-gap states or tune the band structure, extending visible-light absorption; however, excessive defect states may also act as recombination centers. Heterostructure construction further modifies band alignment and interfacial electric fields, promoting directional charge transfer after photoexcitation [104,105,106]. Thus, light absorption, charge generation, and charge separation are not independent processes but are jointly regulated by the nanoscale structure, electronic configuration, and interface architecture of the photocatalyst.

The efficiency of photocatalytic charge generation and transfer is commonly evaluated using ultraviolet–visible diffuse reflectance spectroscopy (UV–vis DRS), photoluminescence (PL), time-resolved photoluminescence (TRPL), transient photocurrent response, electrochemical impedance spectroscopy (EIS), surface photovoltage spectroscopy, and, increasingly, transient absorption spectroscopy. These techniques provide complementary information on light-harvesting ability, recombination probability, carrier lifetime, and interfacial charge-transfer resistance [107]. In this sense, the key descriptors for photocatalysis are not limited to product formation rates, but also include band gaps, CB/VB potentials, apparent quantum yields, carrier lifetime, photocurrent densities, and recombination kinetics. Because photocatalytic rates are highly sensitive to the light source, irradiation intensity, reactor geometry, sacrificial agents, and catalyst loading, meaningful comparison requires reporting the apparent quantum yield, wavelength or spectral distribution, blank controls, and stability tests in addition to product evolution rates.

#### 4.2.1. Separation and Transfer of Photogenerated Carriers

The electron–hole pair recombination is the key to suppressing the efficiency of photocatalysis. A popular method to hinder recombination is creating heterojunction structures, like g-C_3_N_4_/MnO_2_/Pt [108]. Typical heterojunction arrangements include type II, p–n, Schottky, Z-scheme, direct Z-scheme, and step-scheme (S-scheme) heterojunctions [109]. Specifically, various heterojunction designs facilitate the charge separation process through different mechanisms. In classical type II heterojunctions, photogenerated electrons and holes are separated based on their band energies, and thus recombination is suppressed; however, their redox capability may decrease. Moreover, p–n heterojunctions take advantage of the built-in electric field at the interface between p-type and n-type semiconductors for directional carrier migration. Z-scheme and direct Z-scheme systems selectively recombine low-energy carriers and preserve those electrons and holes with higher reduction and oxidation potentials; therefore, redox capability losses related to type II charge separation are minimized. Band bending and internal electric fields play an essential role in S-scheme heterojunctions, thus facilitating interfacial charge separation and preservation of redox carriers.

In recent years, special attention has been drawn to S-scheme heterojunctions, combining the efficiency of charge separation with the retention of redox capability. The application of this concept to tandem photocatalytic processes, including CO_2_ carbonylation, was described [110]. However, it should be taken into account that not only charge separation efficiency but also retained redox capability, interfacial contact, mechanistic evidence, and long-term structural stability should be assessed when designing photocatalytic heterojunctions. Wang et al. outlined ways of enhancing the efficiency of photocatalysis by vacancy engineering and proposed basic rules for optimization [91]. Charge separation efficiency can be measured with PL intensity, carrier lifetime, transient photocurrent response, and electrochemical impedance spectroscopy.

#### 4.2.2. Photocatalytic Surface Redox Reactions

In the context of solar-fuel production, the major surface reduction reactions in photocatalysis include hydrogen evolution and CO_2_ reduction, coupled with oxidative half-reactions such as water oxidation or sacrificial-donor oxidation. Photocatalytic surface redox reactions comprise the hydrogen evolution (HER) and carbon dioxide reduction reactions. The mechanism of photocatalytic HER lies in migrating photogenerated electrons to the active center to reduce H^+^ or H_2_O to produce H_2_. In the carbon dioxide reduction reaction, carbon dioxide gas adsorbs to the surface and is reduced to value-added products, like CO, CH_4_, and CH_3_OH, through a multi-electron process [111]. Product selectivity is dictated by the adsorption arrangement of the intermediate CO_2_ molecules and the charge injection efficiency of the catalyst. Using the photothermal synergism approach, Zhu et al. reported supercharged CO_2_ photothermal catalytic methanation, achieving an unprecedented conversion, rate, and selectivity of the reaction [112].

Table 4 provides a concise comparison of the key electro- and photocatalytic reactions discussed in this section, focusing on their core mechanistic features, representative descriptors, and commonly used performance metrics. This comparison links the reaction-level discussion above with the mechanism-guided catalyst design logic developed in the following section.

### 4.3. Mechanism-Guided Catalyst Design Logic

By studying the mechanisms and descriptors above, the core guiding principle for designing a catalyst can be derived. The performance of the catalyst is determined by how strongly the key intermediate adsorbs on the surface of the material, how readily charge carriers are generated and transferred, and the stability of active sites [87]. These three criteria—adsorption energetics, charge-transfer efficiency, and structural durability—are interdependent, i.e., enhancing one will lead to sacrificing the other. As we shall see later, all enhancement strategies in Section 5 are practical approaches to addressing this problem, e.g., manipulating the electronic structure through doping, generating interface electric fields in heterostructures, or creating defects selective to certain intermediates. This is how mechanistic insight enables scientists to move from empirical practices to rational designs. On the other hand, the mechanisms and descriptors mentioned above are primarily studied under optimized laboratory conditions, and extending them to higher current densities, impure electrolytes, and long-term operating conditions in actual industrial settings still remains a difficult task.

## 5. Performance Enhancement Strategies for Nanostructured Catalysts

**Figure 6:**
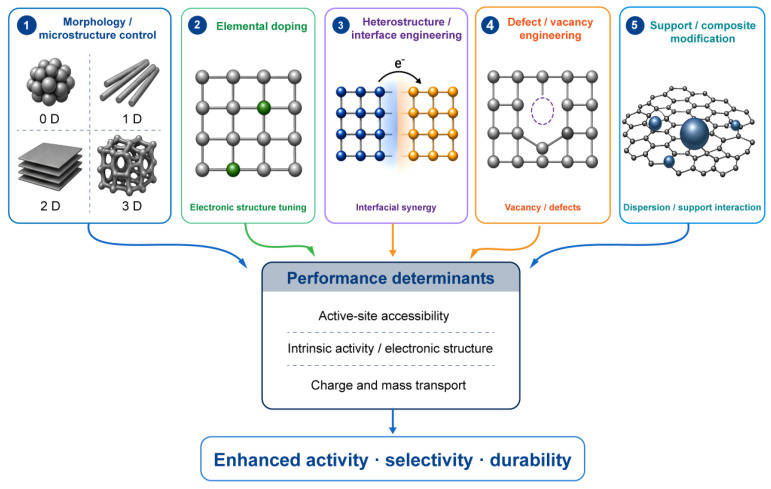
Major enhancement strategies and performance determinants of nanostructured catalysts. Five representative enhancement strategies are summarized, including morphology/microstructure control, elemental doping, heterostructure/interface engineering, defect/vacancy engineering, and support/composite modification. These strategies regulate catalytic performance mainly by improving active-site accessibility, tuning intrinsic activity and electronic structure, and facilitating charge and mass transport, thereby contributing to improved activity, selectivity, and durability.

As mentioned in Section 4, the intrinsic catalytic performance relies on three criteria: the density of active sites, the activity of each site, and the charge/mass transfer efficiency, as supported by case studies showing that active-site modulation can improve catalytic performance [113]. In consequence, performance optimization strategies target one or multiple of these aspects through a mechanistic and descriptor-guided process, as previously discussed. Based on their primary mode of action, the most extensively validated strategies can be classified into five major categories, namely morphology and microstructure control, elemental doping, heterostructure and interface engineering, defect and vacancy engineering, and support/composite modification. This section details the principles, implementation, and representative advances of each strategy, emphasizing how they modulate the key catalytic descriptors to achieve tangible performance gains. The five enhancement strategies illustrated in Figure 6 modulate the active-site density, intrinsic activity, and charge-transfer efficiency, providing a visual guide for mechanism-based catalyst design.

### 5.1. Morphology and Microstructure Manipulation

Morphology and microstructure manipulation are direct strategies for improving catalytic performance since they manipulate the nanoscale morphology and microstructure directly to increase the surface area and expose more active sites [114]. This manipulation primarily targets the first criterion for catalytic performance improvement—the density of active sites—while simultaneously influencing mass transfer. According to spatial dimension, morphological manipulation can be classified into zero-dimensional (0D), one-dimensional (1D), two-dimensional (2D), and three-dimensional (3D) structures. Although 0D nanostructures such as nanoparticles and quantum dots can offer a high specific surface area, they are susceptible to agglomeration when used in practical devices; however, this problem can be partly solved by strategies such as electronic structure manipulation, heterostructure engineering, and elemental doping [115,116,117]. In comparison, 1D structures, including nanowires, nanorods, nanotubes, etc., can facilitate axial electron transport and thereby induce directional charge transfer [118]. For example, self-supporting Co-Ni nanoparticles embedded into nitrogen-doped carbon nanotubes show good OER performance due to their combination of 1D electron transport and 1D active-site arrangement [119]. Meanwhile, self-supporting 3D networks comprising 1D nanostructures, like Pt-Pd-Cu nanowire networks, integrate the benefits of axial transport with robust architectures for glucose electrooxidation [120]. Two-dimensional structures such as nanosheets and nanoplates represent widely studied morphologies for energy-conversion catalysts [121]. Hierarchical 3D structures composed of low-dimensional building blocks preserve the advantages of the latter and, meanwhile, construct continuous porous networks to facilitate diffusion and inhibit agglomeration [122]. The design of nanoporous catalysts for oxygen evolution reaction (OER) has been summarized in an extensive review paper, with particular emphasis placed on active-site exposure [123]. On the other hand, hierarchical nanowall-like MoS_2_ nanosheets grown on bimetallic sulfides exemplify the combined effects of morphology manipulation and synergic active-site arrangement for HER catalysis [124].

As mentioned in Section 3, the morphology of nanostructures can be regulated through various synthesis conditions. For example, ultra-thin NiFe-LDH nanosheets with a thickness of ~2 nm were synthesized through an in situ oxygen plasma-assisted exfoliation approach, thus offering a high electrochemically active surface area and excellent OER performance [125]. Three-dimensional hierarchical porous structures further prevent low-dimensional agglomeration and build mass transfer channels. Nanoporous NiFe-based catalysts with a 3D interconnected structure feature abundant active sites and fast mass-transport behavior, showing good capability for hydrogen generation from urea wastewater under reduced cell voltages [126].

### 5.2. Elemental Doping Engineering

Doping is an atomic-scale strategy for manipulating the electronic structure. Through incorporating foreign elements into the lattice of catalysts, the d-band center of active metal can be adjusted, and intermediate adsorption energies can be fine-tuned, whereas the band structure of semiconductor photocatalysts will be affected [102,103]. Thus, doping aims at enhancing the intrinsic catalytic activity of active sites and is therefore an important approach for modulating intrinsic catalytic activity and, in some cases, alleviating the adsorption-energy scaling constraints that are discussed in Section 4.

Metal doping, non-metal doping, and multi-element co-doping are three general categories. Foreign metals can tune the electronic state of neighboring active sites through charge transfer. Moreover, these dopants themselves can act as catalytic sites for some specific reactions [127]. For example, it is known that iron dopants can tune the binding of oxygen-containing intermediates and improve the OER performance of NiFe-LDH [128]. Non-metal dopants include several types of dopants (N, P, S, and B) that are frequently applied to modify the electronic property and defect status of carbons (g-C_3_N_4_) [129]. As for the co-doping of multiple elements, it combines the advantages of individual dopants, allows for a broader modulation range, and may lead to improved performance, as evidenced by the synergistic co-doping of N and S dopants in carbon materials [130] and Sr and Nb dopants in perovskite ferrites [131].

The versatility of doping is further evidenced across diverse materials and reactions [132,133]. Wang et al. demonstrated that medium-entropy alloy aerogels with modulated d-d orbital coupling can boost pH-general methanol electrooxidation [134], while Cu doping-induced compressive strain in Pd metallene significantly enhances formic acid electrooxidation [135].

### 5.3. Heterostructure and Interface Engineering

It should be noted that heterostructure and interface engineering represent one of the most versatile strategies in the context of electrocatalysis and photocatalysis. The general principle for this strategy includes constructing a composite material with a compatible band structure and electronic property to achieve efficient interfacial charge transfer and dual-site synergy, as well as optimized intermediate adsorption [104,105]. In electrocatalysis, heterostructures manipulate charge distribution at the interface to fine-tune the electronic property of active sites [106]; meanwhile, for photocatalysis, heterostructures can provide an effective route for reducing electron–hole recombination—a critical limitation previously identified in Section 4 [136].

According to the mechanism of charge transfer, heterostructures can be classified into three types, namely type II heterojunctions, Schottky junctions, and step-scheme (S-scheme) heterojunctions. Type II heterojunctions can spatially separate electrons and holes, although this separation is often accompanied by reduced redox capability [137]. At the metal–semiconductor interface, Schottky junctions can efficiently separate electrons through capturing the photogenerated carriers [138,139,140]. S-scheme heterojunctions gain much interest, as they allow effective charge separation while maintaining the capability of charge-carrier redox at the same time, partly addressing the trade-off observed in type II systems [141]. Excellent interfacial contacts are required for all three types, and they can be obtained through methods such as in situ growth, hydrothermal deposition, and atomic layer deposition, as mentioned in Section 3.

The efficacy of heterostructure engineering has been demonstrated in many catalytic systems for photocatalysis and electrocatalysis. In photocatalysis, early investigations on Z-scheme TiO_2_/g-C_3_N_4_ heterojunctions suggest that interfacial defect engineering can enhance hydrogen generation performance [142]. Afterwards, the concept was further advanced for constructing all-solid-state S-scheme homojunctions, wherein an adjustable built-in electric field drives the effective carrier separation [143]; meanwhile, the intrinsic polymerization increases interfacial van der Waals interactions and further facilitates hydrogen evolution and CO_2_ reduction performance [144]. Carbon nitride homojunctions are also applied to couple the hydrogen evolution reaction and the selective benzyl alcohol oxidation reaction [145]. Mechanistically, recent research indicates that localized graphitization in carbon nitride generates a built-in electric field, thus leading to overall water splitting [146]. Also, S-scheme heterojunctions of ZnIn_2_S_4_/H_2_WO_4_ take advantage of the favorable surface electron potential to yield a 4.7-fold enhancement of the hydrogen evolution reaction [147]. From a theoretical point of view, the promotion of water splitting by alkali metal dopants in g-C_3_N_4_ provides further insight into the charge separation mechanism [148]. Electrochemical performance enhancement is another important application area. Intimate interfacial contact in CoSe_2_@NiFe-LDH heterostructures optimizes the adsorption of intermediate species by redistributing interfacial charge, resulting in bifunctional catalytic performance for urea oxidation reaction (UOR) and HER [149]. Meanwhile, Fe_3_S_4_/Ni_3_S_2_ heterostructures facilitate the electrooxidation of ethylene glycol to value-added chemicals and hydrogen [150]; and Ni_3_S_2_/CeO_2_ heterostructures improve OER performance through synergistic interfacial interactions [151].

### 5.4. Defect and Vacancy Engineering

Defect and vacancy engineering is another approach that exploits the surface of nanostructures to introduce controlled defects such as anion vacancies, cation vacancies, lattice distortions, and edge dislocations, thereby increasing unsaturated coordination sites, controlling electronic structure, optimizing intermediate adsorption, and creating defect-related states that can influence carrier separation and recombination [152]. Similar to active-site engineering and adsorption descriptor control, this promising strategy is capable of optimizing both active site density and intrinsic activity and is related closely to the active site and adsorption descriptor concepts outlined in Section 4.

Anionic vacancies, particularly oxygen vacancies, have attracted considerable attention among various defects, mainly because oxygen vacancies are relatively easy to create and can strongly affect catalytic performance. As detailed in Section 3 [153], oxygen vacancies can be generated in metal oxides through hydrogen reduction, plasma etching, annealing in an inert atmosphere, and alkali treatment. Oxygen vacancies can act as adsorption/activation sites, modify the band structure of semiconductor photocatalysts to extend the absorption spectrum, and promote charge separation [154]. In electrocatalysis, cation vacancies are also important for manipulating electronic structure and catalytic activity [155].

A wide range of catalytic reactions can benefit from defect engineering. For example, the coupling effect of photo-induced oxygen vacancy generation and surface heterostructure reconstruction has been demonstrated to promote CO_2_ reduction in BiOBr-based photocatalysts [156]. The role of oxygen vacancies in metal oxides in photocatalytic CO_2_ reduction has been comprehensively reviewed by Jiang et al. [157]. It describes methods of synthesis, characterization, and mechanistic function of oxygen vacancies in promoting light absorption, charge separation, and surface CO_2_ activation. The atomic defect structure was identified as an important factor in tuning the n-π* excitation and charge separation, thereby improving the efficiency of photocatalytic hydrogen production and CO_2_ reduction in g-C_3_N_4_ [158]. Zuo et al. summarized how defect engineering can be applied to optimize photocatalytic CO_2_ reduction [159]. For electrocatalytic reactions, it has also been found that oxygen vacancy engineering is an efficient way to manipulate the electronic structure of transition-metal oxide and enhance their intrinsic activity in OER and HER processes [152,154].

However, defect engineering must not be viewed as a principle in which a higher defect content results in improved catalytic behavior. The catalytic function of defects is highly dependent on the nature of the defects, their concentration, distribution, and local environment. Properly introduced oxygen vacancies or surface defects can create unsaturated sites, modify the density of states close to the Fermi level, improve the adsorption of reactants and the activation of CO_2_, H_2_O, O_2_, or oxygenated species. Defect-induced states can increase visible-light absorption and charge separation in photocatalysis. However, an excessively high concentration of defects can serve as recombination centers and induce photocorrosion or decrease stability. In electrocatalysis, defect-rich surface states can optimize intermediate adsorption and lower kinetic barriers. However, metastable defect sites can be partially healed, reconstructed, or dissolved upon prolonged use of the catalysts. Therefore, future research in defect engineering should go beyond the qualitative description like “higher defect concentration” and determine the defect concentration quantitatively, define the actual defect state of the active site, and relate defect changes to the activity, selectivity, and stability of the material. A recent study on Mo–CeO_2_ photocatalysts supported on a structured scaffold demonstrates that defect engineering and support engineering can cooperatively enhance CO_2_ photoconversion [160].

### 5.5. Support and Composite Modification Engineering

The purpose of support and composite modification engineering is to optimize the overall performance, especially durability, of catalysts. The key concept behind it is the immobilization of the active phase on a proper support, thus suppressing nanoparticle agglomeration, improving conductivity, accelerating charge transfer, and maximizing the accessible surface area [161,162]. This strategy focuses on the third performance factor, i.e., charge-transfer efficiency and operation durability, which is crucial for practical applications.

Graphene, carbon nanotubes, carbon cloth, and MOF-derived porous carbon are among the most widely used supports, characterized by a high specific surface area, good electrical conductivity, and favorable chemical stability, which can facilitate uniform dispersion of the active component and fast electron transport [163,164]. Apart from carbon, metal foams (such as Ni, Cu), conductive polymers, and porous oxides can also act as supports. In a self-supported electrode structure, the active phase directly grows on the conductive substrate without using a polymer binder, hence minimizing the contact resistance and improving stability [165,166].

The advantages of support engineering have been demonstrated comprehensively in the design of powder-supported and self-supported electrocatalysts. Dai et al. dispersed MoS_2_ nanoparticles on reduced graphene oxide and suppressed the agglomeration of the active phase, as well as improved conductivity, and achieved high HER performance [167]. Liu et al. uniformly distributed (NiFe)S_2_ nanoparticles on graphene nanosheets and realized a high-efficiency and stable OER due to fast charge transfer [168]. Liang et al. deposited Co_3_O_4_ nanocrystals on reduced graphene oxide and showed that the carbon support could prevent nanoparticle agglomeration and deliver long-term cycling stability comparable to that of Pt-based benchmarks under the tested conditions [169]. Meng et al. grew Ni_3_S_2_ nanosheets directly on the Ni foam substrate to construct a highly efficient and robust self-supported bifunctional electrode in the urea oxidation and hydrogen evolution reactions [170].

### 5.6. Summary of Performance Enhancement Strategies

As mentioned above, the five strategies discussed here are not independent of each other. In real cases, these strategies can be implemented in combinations to achieve a comprehensive enhancement of performance. For example, morphology engineering combined with heteroatom doping and defect engineering could effectively increase active-site density, intrinsic activity, and charge-transfer rate, therefore leading to synergistic performance enhancement in suitable systems. Most importantly, the same strategy usually operates through different mechanisms in electrocatalysis and photocatalysis. Elemental doping, for instance, adjusts the d-band center and adsorption energy of intermediates in electrocatalysis, but it also controls the band gap and charge separation efficiency in photocatalysis. While heterostructure engineering optimizes interfacial charge redistribution and may alleviate scaling-relation constraints in electrocatalysis, it plays the role of suppressing electron–hole recombination in photocatalysis through energy band alignment. To some extent, recognizing their dual nature not only broadens the mechanistic understanding but also expands the application scope of the strategies. For example, the design of heterogeneous structures optimized for efficient charge separation in photocatalysis could be modified and adopted in electrocatalytic systems after certain adjustments, and vice versa. Nevertheless, it is important to remember that a key evaluation criterion for a strategy is not only enhanced activity but also performance durability under realistic operating conditions. As explained in Section 7, durability during long-term operation and scalability of the fabrication process are the main criteria for screening out useful strategies from theoretical study. All these strategies rely on the knowledge of the catalytic mechanism and provide guidelines for designing nanostructured catalysts. The optimized materials resulting from these approaches have found wide application in diverse clean energy-conversion scenarios, which are the subject of the next section.

Table 5 provides a concise comparison of the five enhancement strategies in terms of their structural targets, mechanistic effects, performance benefits, and potential trade-offs. This summary serves as a bridge between mechanism-guided catalyst optimization and the application-oriented discussion in the next section.

Importantly, these enhancement strategies also involve trade-offs that are sometimes underestimated in activity-centered studies. Increasing the surface area may expose more active sites but can also accelerate corrosion, dissolution, or structural collapse. Doping and defect engineering can optimize intermediate adsorption and charge separation, but excessive dopants or defect states may create inactive sites, recombination centers, or unstable coordination environments. Heterostructure and interface engineering can promote charge transfer and multifunctional catalysis, yet poorly controlled interfaces may introduce additional resistance or become unstable during reconstruction. Support and composite modification improve dispersion and conductivity, but weak coupling between the active phase and the support can lead to leaching, detachment, or loss of active sites. Therefore, a strategy should be evaluated not only by its ability to improve apparent activity, but also by whether the resulting structure remains identifiable, stable, and reproducible under realistic operating conditions.

## 6. Applications of Nanostructured Catalysts in Clean Energy Conversion

Strategies of enhancing catalytic performance, including controlling morphology and doping, as well as forming heterostructures and engineering defects, are rarely practiced separately. When they are carefully used together, the activity, selectivity, and durability of nanostructured catalysts can be efficiently optimized to reach those needed for commercial application [171]. Based on the discussion in Section 4 and Section 5, this section introduces recent advances in representative fields, including electrocatalytic and photocatalytic water splitting for hydrogen production, CO_2_ reduction and valorization, fuel cells and metal–air batteries, and energy-saving electrolysis for the production of valuable chemicals. The focus throughout is on catalysts that have been systematically optimized using the strategies of Section 5, with emphasis on their performance under application-relevant conditions.

### 6.1. Electro-/Photocatalytic Water Splitting for Hydrogen Production

Hydrogen, with an energy density of 142 MJ kg^−1^, is a promising carbon-free fuel that is considered for the future of sustainable energy sources [172]. Water splitting, powered by either renewable energy or sunshine, has been recognized as the most feasible pathway to produce green hydrogen, with essentially zero greenhouse gas emissions under ideal conditions [173,174,175]. The remaining core challenge is the development of efficient bifunctional catalysts that are capable of driving both the HER at the cathode and the OER at the anode with low overpotential, high efficiency, and good durability [176].

In electrochemical water splitting, the nanostructured catalysts, optimized via controlling the morphology, constructing heterostructures, and manipulating defects, can achieve performance comparable to or exceeding that of commercial noble-metal benchmarks under specific alkaline conditions. In contrast, the strategies of regulating the electronic structure are extensively explored in acidic OER [177,178,179,180,181,182,183,184,185,186]. Fu et al. designed the medium-entropy NiCoFeP@NiCoFe-LDH heterostructure, obtaining a cell voltage of 1.42 V and a long-term stability of 600 h at 500 mA cm^−2^ [187]. Wang et al. fabricated ternary phosphide Mn-Co-Fe nanosheets on Ni foam to obtain a 1.66 V cell voltage at 100 mA cm^−2^ [188]. Additional representative examples include MoS_2_/Au^0^/N-CNT from Au recovery for HER [189], and Cu_3_P-CoP heterostructures with cation vacancies for high-current-density HER [190], and several theoretically predicted two-dimensional materials, such as monolayer In_2_Te_5_ [191] and CuP_2_Se [192], as photocatalysts for solar water splitting.

In photocatalytic water splitting, there are steady advances in nanostructured catalysts via element doping, constructing heterostructures, and introducing defects to increase solar-to-hydrogen conversion efficiency [193]. The critical task is to enhance electron–hole separation while ensuring high redox capacity in the photocatalysis. To solve this issue, the heterojunction strategy described in Section 5 is adopted in the S-scheme [194]. Ding et al. reported the fluorone-based covalent triazine framework (CTF)/twinned Zn_0.5_Cd_0.5_S S-scheme heterojunction that exhibited a photocatalytic H_2_ evolution rate of 247.62 mmol g^−1^ h^−1^ [195]. Shi T et al. built an S-scheme heterojunction based on carbon nitrides to increase hydrogen production efficiency and perform selective benzyl alcohol oxidation simultaneously [196]. Ferrando-Ferrero et al. demonstrated V-doped Ti-squarate MOFs that could efficiently carry out photocatalytic overall water splitting to evolve H_2_ and O_2_ simultaneously under artificial sunlight without any additional cocatalyst [197]. These examples underscore the broad potential of nanostructured catalysts in solar hydrogen generation. Nevertheless, photocatalytic H_2_ evolution, in the presence of sacrificial electron donors, selective organic oxidation coupled with H_2_ production, and true overall water splitting, should be distinguished carefully because they differ substantially in thermodynamic difficulty, charge-balance requirements, and practical relevance. For overall water splitting, simultaneous and stoichiometric H_2_/O_2_ evolution, long-term stability, and catalyst robustness without sacrificial reagents or noble-metal co-catalysts are particularly important benchmarks.

### 6.2. Electro-/Photocatalytic CO_2_ Reduction and Valorization

The continuously increasing level of atmospheric CO_2_ requires more action besides reducing CO_2_ emissions. Transforming atmospheric CO_2_ into chemicals or fuels such as CO, CH_4_, HCOOH, and C_2+_ is not only effective in reducing carbon emissions but also serves as a storage of renewable energy [198]. One key issue is how to develop highly efficient catalysts with strong selectivity and durability to activate inactive CO_2_ molecules for further reduction reaction [199].

In photocatalytic CO_2_ reduction, there are many examples that use nanostructured catalysts optimized by regulating the band structure, forming heterojunctions, engineering defects, and applying co-catalysts [25,200]. Several recent review articles summarize the progress of indium-based sulfides [26], metal/semiconductor photocatalysts for CO_2_ hydrogenation [201], bismuth-based compounds [202], and BiOX (X = Cl, Br, I) for photocatalytic CO_2_ reduction [203]. Additionally, there are some molecular complex photocatalysts, like Ca(II) [204] and dinuclear Gd(III) [205], showing high selectivity for CO production from CO_2_ reduction. Yuan et al. reviewed the advances of nickel-based nanostructured catalysts for photothermal CO_2_ hydrogenation [206]. Specifically, Ding et al. designed Ru-doped Ni/ZrO_2_ and found its good performance in photocatalytic CO_2_ methanation with a CH_4_ selectivity up to 100% [207]. Gao et al. developed the synergy of surface frustrated Lewis pairs and CuPt alloys to improve the activity of photocatalytic CO_2_ hydrogenation [208].

For electrocatalytic CO_2_ reduction, there are also many representative works demonstrating the selectivity of CO_2_ reduction to specific products by optimizing the catalysts’ morphology and interfaces [209]. For instance, Yang et al. designed a Ag/Cu_2_O heterostructure to selectively produce C_2+_ species, with a Faradaic efficiency of 77.8% at a current density of −300 mA cm^−2^ in flow cells [210]. Wei et al. achieved a 99% Faradaic efficiency of formate production at a current density of 2 A cm^−2^ via Cl-doped SnO_2_ nanoflowers and maintained its stability up to 520 h at a current density of 3 A cm^−2^ [211]. Xu et al. successfully produced C_2_H_4_ from CO_2_ reduction with a Faradaic efficiency of 54.7% at a current density of 600 mA cm^−2^ under acidic conditions (pH = 1) on a Cu nanostructure with cavity-network architecture [212]. It is clear that CO_2_ reduction via optimized nanostructured catalysts has great potential in closing the carbon cycle. In addition, other multi-electron reduction reactions, including nitrate electroreduction to ammonia, where promising single-atom catalysts have been identified through theoretical screening [213], as well as N-doped Co_3_O_4_ nanowires with high faradaic efficiency [214], nitrogen reduction on curvature-tuned FeN_4_ carbon sites [215], and photocatalytic ammonia synthesis [216], also indicate that the nanostructured catalysts designed in the present work can be broadly applied.

### 6.3. Fuel Cells and Metal-Air Batteries

Fuel cells and metal–air batteries are highly efficient energy-conversion devices that are capable of directly converting chemical energy to electricity with high theoretical energy density and zero or near-zero emissions [217,218]. In both cases, the oxygen reduction reaction (ORR) is the critical process and rate-limiting step, and thus, efficient ORR catalysts play a crucial role [219].

As one example, the substitution of expensive and scarce Pt/C catalysts in fuel cells by earth-abundant materials has become the major driving factor for the development of ORR catalysts in the past two decades [220]. Nitrogen-containing transition-metal single atoms supported by porous carbon materials (M–N–C, M = Fe, Co, Mn) are considered as leading candidate catalysts for ORR. For instance, the Fe–N–C electrocatalyst has only a 20~30 mV difference in the half-wave potential compared with the Pt/C catalyst in an acidic medium and performs better than Pt/C in an alkaline electrolyte. In addition, the Fe–N–C catalyst has much higher methanol tolerance and resistance to carbon monoxide (CO) poisoning than the Pt/C catalyst [221]. Macedo Andrade et al. reported the catalyst constructed based on the MOF-derived Co/Cu-embedded N-doped carbon, which can perform well in the ORR, OER, and HER in a trifunctional way [222].

However, different from fuel cells, rechargeable metal–air batteries need bifunctional catalysts that are capable of simultaneously driving the ORR and OER with a minimum energy difference between charge and discharge to realize a reversible cycle [223]. To measure the bifunctionality of the catalysts, the difference between oxygen evolution reaction (OER) potential and ORR half-wave potential of the bifunctional catalyst is measured at the current density of 10 mA cm^−2^. A value smaller than 0.7 V is defined as “excellent,” while that of 0.7~0.9 V represents a state-of-the-art performance [224]. Representative progress includes the transition-metal-based heterostructure catalysts (e.g., Co/CoO_x_, NiFe-LDH, and carbon); the perovskite oxides with tunable B-site cations; and the composite based on MOFs to combine the active component for both ORR and OER in a single nanostructure [225]. Examples include the Fe–N–C catalyst with a Δ*E* of 0.63 V, constructed by Tu et al., and this catalyst showed an ultra-high Zn-air battery peak power density of 218 mW cm^−2^ and cycling ability for over 200 h [226]. As another example, alloy-based strain engineering also offers a new solution. Specifically, the PtCu alloy aerogel has been proven to exhibit outstanding ORR activity via strain engineering with mass activity, up to 1.65 A mgPt^−1^ [227]. Furthermore, Zhang et al. found that Cu–Co dual single atoms anchored on nitrogen-doped carbon nanotubes showed comparable ORR activity to Pt/C catalysts with much better durability [228]. Zhong et al. designed single cobalt atoms supported on Co_3_O_4_ and nitrogen-doped activated carbon and demonstrated a good Δ*E* of 0.77 V for a rechargeable Zn-air battery [229].

These advances, built on the heteroatom doping, defect engineering, and support modification strategies of Section 5, position nanostructured non-noble-metal catalysts at the threshold of commercial viability for fuel cells and metal–air batteries.

### 6.4. Energy-Saving Electrolysis Systems and Beyond

In addition to increasing the activity of the catalysts discussed in Section 5, the strategies described therein may also be used in the development of energy-saving electrolysis systems, which can produce valuable chemicals while saving energy. By using more thermodynamically favorable oxidation reactions (such as urea oxidation (UOR, 0.37 V vs. RHE), hydrazine oxidation, or alcohol oxidation) in place of a slow OER (1.23 V vs. RHE), the energy-saving hydrogen generation system with wastewater treatment or value-added chemical production can be realized [230,231]. Recent studies on alcohol electrooxidation include the following: glucose electrooxidation on PdRu-based sites [232]; ethanol oxidation on PdMoW trimetallene [233]; morphology-controlled Pt nanocrystals [234]; methanol and formic acid oxidation on defect-rich PtCuMn nanoframes [235]; and ethanol oxidation on sub-10 nm PdNi@PtNi core/shell nanoalloys [236].

Among the mentioned hybrid electrolysis systems, the most well-researched one is urea-assisted water electrolysis due to the widespread occurrence of urea-containing wastewater and substantial voltage savings by using UOR in replacement of the oxygen evolution reaction [237]. By using phosphorized CoNi_2_S_4_ yolk–shell spheres, a cell voltage of 1.402 V was achieved at 10 mA cm^−2^ for urea electrolysis, with a durability of over 100 h in a work reported by Lu et al. [238]; Guo et al. reached 100 mA cm^−2^ at 1.475 V and stability over 500 h via a Ni_2_P_4_O_12_/NiTe heterojunction, which drives the UOR via a rapid Ni^3+^/Ni^2+^ transformation process [239].

Another important type of electrolysis is seawater electrolysis, which has become a strategic alternative to freshwater-based hydrogen production, especially suitable for arid coastal regions. The issue to solve, in this case, is the lack of stable catalysts against the chlorine evolution reaction due to the high chloride content in seawater; recent review articles about solving this issue include the use of a porous structure, a Cl^−^ blocking layer, and wettability manipulation [240,241]. Notable works in this field include chlorine-free seawater splitting via a tip-enhanced Co_3_S_4_ catalyst at 100 mA cm^−2^ for over 500 h [242]; a long-lived seawater electrolysis system with over 2000 h durability at 500 mA cm^−2^ [243]; and seawater hydrogen production catalyzed by hydrazine through an Fe-Co_2_P/CeO_2_ heterostructure [244].

These extended applications underscore the broad potential of nanostructured catalysts beyond conventional energy-conversion systems, pointing toward integrated, energy-efficient, and environmentally beneficial electrochemical technologies.

### 6.5. Summary of Application Achievements

Over the last few decades, nanostructured catalysts have been successfully transferred from the basic research stage to the demonstration stage of various clean energy-conversion processes. The strategies described above have been applied in real cases in order to optimize their performances, connecting understanding and rational design, performance enhancement, and practical application. In particular, there are differences in optimizing the goals of electrocatalytic and photocatalytic systems with respect to a certain strategy: the main function of heterojunctions is charge redistribution in electrocatalysis, while heterojunctions in photocatalysis should meet the requirement of energy band gaps. Design of high-performance catalysts should therefore be conducted based on driving force and chemical reactions’ characteristics.

The major application scenarios discussed in this section and their corresponding performance metrics and practical requirements are summarized in Table 6. However, in spite of the above achievements, most of the demonstrations are still carried out at a laboratory scale; as far as most catalysts reviewed here are concerned, a key bottleneck for industrial implementation is often durability rather than intrinsic activity. This fact starts to change the orientation of the research now, and in Section 7, we will discuss the issues in practical implementations that must be addressed before commercialization.

To complement the application-level comparison in Table 6 and provide a clearer quantitative perspective on the state-of-the-art, selected representative nanostructured catalysts and their reported benchmark performance are summarized in Table 7. These examples compare catalyst systems according to the reaction type, structural design features, key activity metrics, selectivity or conversion metrics, stability, and operating conditions. They illustrate the substantial progress achieved across water splitting, CO_2_ reduction, oxygen electrocatalysis, metal–air batteries, and energy-saving electrolysis, while also highlighting the diversity of testing conditions and durability protocols used in the literature.

The examples presented above should be considered as benchmarks, indicating achievements in the field rather than as a direct ranking of catalyst performance. In addition to the differences in driving force, reactor configuration, electrolyte/feed composition, light intensity, catalyst loading, electrode architecture, and durability testing procedures, the electrochemical and photocatalytic systems differ in many other aspects, making the activity results non-transferable between applications. In other words, catalysts that provide outstanding results when tested under optimal laboratory conditions will not necessarily show such advantages when operated under high-current density, concentrated reactant streams, long-term illumination, the presence of impurities in the electrolyte, or in integrated devices. It is also crucial to consider the stability and reproducibility of the experiments, the availability of materials, the scalability of synthesis, and compatibility with real reactors/devices.

## 7. Current Challenges and Future Prospects

Nanostructured catalysts have witnessed remarkable success in laboratory experiments and prototypes, and some of them have come close to the performance of noble-metal benchmarks or even surpassed them under certain experimental conditions [217], as explained in Section 6. However, for practical application, apart from having high intrinsic activity, it is necessary for the catalysts to function efficiently at high-current density, under continuous illumination, concentrated or impure-feed streams, and complex mass-transport conditions. The issues associated with photocatalysis are low apparent quantum efficiency, charge-carrier recombination, photocorrosion, and catalyst recovery/reuse. For the electrocatalytic systems, major obstacles include durability at high-current densities, tolerance to poisoning, integration with electrodes, and scalability of fabrication. Therefore, this section is dedicated to the main challenges preventing laboratory performance of catalysts from practical realization.

### 7.1. Core Challenges in Practical Applications

#### 7.1.1. Mechanistic Ambiguity and the Elusive Nature of Active Sites

A key challenge is that the structures and active states of these catalysts under operating conditions are often not fully understood. Most of our mechanistic understanding has come from characterizing the catalyst after it has been removed from the reactor and developing theoretical models using idealized computer simulations of how they should behave. While these are both good sources of information, neither of them captures how much the catalyst can change (i.e., through dynamic restructuring, phase transitions, and/or site leaching) at higher current densities or after prolonged periods of operation [245]. Additionally, with heterostructured materials that comprise two or more components and thus have two or more types of active sites, even finding the answers to basic questions (i.e., the exact locations of active sites and the interactions between different components of a heterostructured catalyst) remains a matter of ongoing scientific debate [177]. Consequently, without clearer operando-based information on catalyst surfaces, rational catalyst design will still involve considerable uncertainty despite advances in characterization and modeling [246].

#### 7.1.2. The Lab-to-Industry Performance Disparity

A second difficulty is the disparity between testing catalysts in the laboratory and their subsequent application in an actual device. Most academic studies report results from half-cell experiments: typically having a current density of 10 to 100 mA/cm^2^; utilizing only clean, dilute electrolytes; starting with pure reactants; and conducting testing for only an extremely short duration (less than 10 to 30 h). In contrast, industrial electrolysis occurs at current densities well in excess of 500 mA/cm^2^, usually employing concentrated or impure feedstocks, operating under elevated temperatures and pressures, and requiring an operational stability of 1000 to 5000 h (without maintenance). Consequently, testing protocols to systematically benchmark catalysts and electrolysis systems under these types of conditions are still lacking [29]. Moreover, standardized durability test procedures to measure operations at industrial current densities are just starting to be developed [20]. This performance gap has stalled many otherwise promising materials at the laboratory stage and remains a persistent obstacle to translating fundamental discoveries into practical applications.

#### 7.1.3. Scalable, Cost-Effective Synthesis

Another issue is manufacturability. A vast majority of the state-of-the-art catalysts introduced in Section 3 and Section 5 are synthesized using laboratory batch methods such as hydrothermal processing, atomic layer deposition, and precise interfacial assembly. Although such approaches afford high structural precision, they lack scalability. Lab-scale synthesis yields small batches, limited yields, and fine-tuning of nanostructure, defect density, and interfacial quality does not survive scale-up to reactor-size processes, leading to significant variability between batches. This is a severe problem for any technology demanding consistency in specifications. The cost is another limiting factor. Even in the case when all constituent elements are earth-abundant, expensive synthesis can drive the price of the product beyond competitiveness against industry-standard catalysts. Therefore, there is a need for simpler and more environmentally friendly synthetic approaches that are capable of producing kilograms of active material in a scalable manner without sacrificing structural quality [20].

#### 7.1.4. Durability in Real Conditions: Stability and Tolerance to Poisoning

Another crucial problem is the lack of sufficient stability of catalysts under practical conditions of operation. Laboratory tests for stability are carried out in purified electrolytes or streams with controlled composition within several tens of hours only, while the practical system requires stable operation in the presence of impurities, fluctuating local environments, and accumulated reaction products. For instance, the presence of chloride ions in seawater electrolysis, trace CO in reformate streams, sulfur- or nitrogen-containing impurities, and products formed during extended operation can gradually poison active centers or modify surface reaction channels. Aside from chemical poisoning, catalysts can suffer from structural degradation, including dissolution of active sites, metal leaching, surface oxidation or reduction, phase modification, aggregation, carbon or support corrosion, catalyst detachment, and loss of metal-support interaction [247]. It was shown by Wang et al. that the CO tolerance of PtRu alloys in methanol oxidation can be improved by adjusting the Pt/Ru atomic ratio, demonstrating the role of active-site regulation in the development of poisoning-resistant catalysts [248].

In the case of photocatalytic systems, one also needs to consider additional deactivation mechanisms like photocorrosion, irreversible oxidation or reduction in the surface, as well as the healing of defects, cocatalyst detachment, fouling of the surface, and a progressive decrease in charge-transfer efficiency at the interface under illumination. All these phenomena can modify the nature and distribution of the active centers during the operation process and thus make initial activity measurements insufficient for evaluating the practical feasibility of catalysts. That is why the evaluation of stability needs to include both long-term testing under realistic conditions and subsequent characterization of the sample after reaction and in operando in order to evaluate the cause of the retained activity, whether it is structural stability or dynamic reconstruction.

#### 7.1.5. Catalyst Deployment Mode and Photocatalyst-Specific Stability Issues

Aside from the inherent stability of the catalyst, the way in which it is used also plays an important role in determining the practical feasibility of the catalytic system. Many photocatalytic studies use dispersed slurry systems since these systems allow reaching maximal contact between the catalyst and reactant, as well as sufficient light exposure. However, there are serious problems with recovery, separation, and the reuse of the catalysts in such systems. Catalyst particles can be irreversibly lost during recycling, cause secondary contamination, and aggregate during long-term usage. This problem becomes especially acute for earth-abundant photocatalysts, which are still subject to gradual deactivation of active centers, photocorrosion, and low apparent quantum efficiency due to fast charge-carrier recombination. Similar limitations have been emphasized recently in a review of ZnO-based heterostructure photocatalysts for environmental applications [249].

Immobilized photocatalyst films, coatings, monoliths, membranes, and structured reactors represent possible alternatives to slurry systems. These approaches facilitate catalyst recovery and integration into reactors, but impose additional restrictions related to reduced accessible surface area, limited mass transport, weak light penetration, poor coating adhesion, and an inability to achieve uniform catalyst loading on a large scale. Thus, the task is not only to find highly active catalysts in powder form, but also to select the most suitable catalyst configuration. An evaluation of photocatalysts in the future needs to include not only the rates of product formation and apparent quantum yields, but also catalyst recovery efficiency, activity retention after cycles, coating adhesion, leaching behavior, and stability under illumination.

### 7.2. Future Directions and Outlook

Meeting these challenges will require concerted effort on several fronts.

#### 7.2.1. Operando Studies and Machine Learning-Guided Design

To close the gaps identified in Section 7.1.1, efforts have to be directed towards the operando characterization of catalyst behavior. Various forms of in situ and operando spectroscopy, including Raman, X-ray absorption, IR, and environmental transmission electron microscopy (TEM), enable the monitoring of catalytic reactions in real time [250]. Experimental measurements have to be supplemented with first-principles modeling; operando studies combined with DFT already constitute a powerful tool for determining active-site identities and kinetics [251]. At the same time, computer-aided methods like machine learning and high-throughput screening are increasingly applied to explore catalyst composition–structure–performance relations in a large parameter space [252]. As machine learning algorithms rely on large amounts of quality experimental data, building dedicated databases will be crucial to make this approach viable for catalyst development. It should incorporate experimental data in a standardized fashion.

#### 7.2.2. Industrial-Oriented Catalyst and Device Co-Design

The performance gap found in Section 7.1.2 suggests the need to revise the criteria for assessing the performance of catalysts. The main goal of further catalyst design is not to enhance the efficiency of ideal half-cell reactions, but to create catalysts with active sites and electrode configurations that ensure high stability and activity of a catalyst at current densities used in industry (≥500 mA cm^−2^) [30]. Additionally, catalyst layers should be compatible with membrane electrode assemblies, gas-diffusion electrodes, flow cells, current collectors, and flow-field designs affecting mass transport, bubble removal, local pH gradients, and ohmic losses. Self-supported electrodes without polymeric binders represent one possible solution to increase the mechanical stability and charge transfer at high currents [22]. Likewise, gas-diffusion electrodes with tunable wettability and porosity are a way to increase CO_2_ conversion under industrially relevant conditions [253]. Consequently, increased coordination between catalyst synthesis, electrode fabrication, electrolyte design, and electrolyzer engineering, along with testing in accordance with industrial criteria, will be required for the implementation of breakthroughs achieved in the laboratory.

For photocatalytic systems, an industrial-oriented co-design should also consider the mode of catalyst deployment and photoreactor setup. Slurry reactors, immobilized films, membrane-supported catalysts, and monolithic photocatalysts should be assessed using different performance and durability metrics and cannot be directly compared in terms of the reaction rate. Photoreactor design, depth of light penetration, catalyst loading, optical path length, and gas–liquid–solid contact greatly influence the apparent reaction rate, which should be considered in addition to catalyst properties. For carbon-based electrocatalysts, future research should be focused on carbon corrosion, stability of active sites, stability of heteroatom doping, reproducibility of the pore structure, and incorporation of catalysts into large-area electrodes. The discussed factors show that industrial translation involves a catalyst–electrode–reactor co-design rather than an evaluation of powder catalysts, slurries, or electrodes with a small surface area.

#### 7.2.3. Green, Scalable, and Economical Synthesis Routes

As mentioned above, the scaling issues require shifting from laboratory batch synthesis to continuous industrial-scale production that should provide high-throughput and good reproducibility, with preservation of structural control [31]. It is crucial to achieve batch-to-batch consistency in the case of nanostructured catalysts since even small variations in composition, defect density, particle size, porosity, and interface structure may lead to considerable variations in catalytic activity. In-line process analytical technologies and feedback control systems can be used to control synthesis process parameters and to achieve higher reproducibility. In addition, synthesis design should take into account the life-cycle considerations, such as the recyclability of critical elements, the environmental impact of precursor chemicals, solvent consumption, energy input, and waste disposal [32,254]. Thus, green and scalable synthesis is not only desirable but also necessary for the commercial viability of the developed catalysts.

Within this framework, catalyst costs should be assessed not only as the price of the active elements. Manufacturing costs also depend on the availability of precursors, solvent consumption, synthesis time, pressure or temperature conditions, template removal, pyrolysis or phosphiding, post-treatment, yield of product, and batch reproducibility. Using techno-economic analysis, it can be determined whether the improved catalytic activity warrants additional manufacturing complexity, whereas life-cycle assessment can be used to compare the environmental impact of different synthesis and catalyst deployment processes. Both types of analysis are especially useful for nanostructured catalysts that are obtained through multistep synthesis, high-temperature treatment, low-yield synthesis routes, complicated support materials, or scarce elements.

#### 7.2.4. Structural and Compositional Solutions for Stability and Poisoning

As outlined above, durability remains challenging for electrocatalysts and requires a solution in terms of structure and composition. Protection against sintering, metal-support corrosion, and leaching can be ensured by protective carbon coatings, creating strong metal–support interactions and designing stable interfaces between heterogeneous phases [255]. Apart from structural stabilization, active sites have to be engineered to withstand chemical poisoning. For example, the presence of chloride ions can poison catalysts in the process of seawater electrolysis; CO traces in reformate and urea in electrolytes can gradually poison active sites of corresponding catalysts. Other than stabilizing the structure, modifying the surface and tuning the composition can endow the catalyst with resistance to typical poisons, like chloride ions from the electrolysis of seawater or CO from reformate gas streams, as well as the accumulation of unwanted products such as ammonia from urea electrolysis, so that the catalysts may operate stably under feedstocks that contain these toxicants without significant performance loss [256]. Catalysts combining high intrinsic activity with robust long-term stability are more likely to be suitable for industrial applications.

### 7.3. Concluding Remarks

Advancements in the area of nanostructured catalysts for clean energy conversion have been enormous within the last two decades. However, there still exist a number of obstacles preventing further transition from research into industry. Challenges include a poor mechanistic understanding of catalyst dynamics under realistic operational conditions, insufficient performance at commercially relevant current densities, non-scalable and cost-ineffective synthesis routes, and insufficient stability in the face of impurities. Altogether, such issues highlight the field that has entered a new phase: the activity of these catalysts is no longer a major bottleneck; however, proving activity in real-world systems becomes the focus of future developments. Despite being very challenging, these tasks are solvable; the combination of improvements in operando characterization, data-driven catalyst discovery, large-scale synthesis methods, and device-level engineering is sufficient to bring us closer to commercial implementation. Hence, nanostructured catalysts may serve as important components for future carbon-neutral energy technologies.

## 8. Conclusions

Among the major points made in this review is that the development of nanostructured catalysts for electro- and photocatalytic energy-conversion applications has reached a crossroads. In many cases, as described in Section 5 and Section 6, where reactions have been investigated during the past few decades, such as alkaline water splitting, urea oxidation, oxygen reduction, and selected CO_2_ electrocatalysis pathways, activity is no longer the only key limiting factor. Indeed, earth-abundant nanostructured catalysts have achieved or approached precious metal performance under optimal laboratory conditions. In the case of photocatalysis, heterojunction design, defect management, and cocatalyst design have helped to improve charge separation, light harvesting, and selectivity towards products. The key question now becomes the demonstration of high performance of catalysts under realistic operating conditions, i.e., with high-current densities, reactant impurities, long-term operation, prolonged illumination, and realistic reactor/device configuration.

These developing challenges pose new requirements for the study of catalysts. The techniques considered in this review, which are morphology control, elemental doping, heterostructure and interface design, defect and vacancy engineering, and support engineering, constitute a fully developed set of techniques, allowing tuning of catalyst activity. Yet, further optimization and incremental improvements in overpotential or the reaction rate will hardly help accelerate adoption. What needs to be shown is that these performance improvements can persist during transfer from rotating disk electrode measurements to membrane electrode assembly configuration, from purified electrolytes to real water, from one-off experiments to long-term operation, and from dispersed photocatalyst powder to slurry or film configurations.

To address this problem, it is necessary to consider the catalyst development and implementation in a broad context, as discussed in Section 7. Future studies have to devise realistic testing protocols for catalysts with high-current density and illumination, establish scalable synthetic procedures, elucidate the dynamics of active-site evolution via operando techniques, and quantify catalyst performance, not just in terms of activity and the apparent reaction rate but in terms of activity, selectivity, stability, operating conditions, and the implementation method. Early techno-economic analysis, life-cycle assessment, material availability, and end-of-life issues need to be addressed during catalyst development. This is where the real value of nanostructured catalysts for sustainable energy conversion and carbon-neutral development will be revealed.

## Figures and Tables

**Table 1:** Positioning of the present review relative to representative previous reviews on nanostructured catalysts for energy conversion.

Previous Review Focus	Representative References	Main Scope and Strength	Remaining Limitation	Positioning of the Present Review
General nanoscale catalyst engineering	[7,9]	Discusses nanoscale control of active sites, surfaces, interfaces, selectivity, and stability.	Not specifically organized around electro-/photocatalytic energy conversion and practical translation.	Applies nanoscale design principles to electro- and photocatalytic energy conversion within a design-to-application framework.
Single-atom and dual-atom catalysts	[13,14,15]	Focuses on isolated or paired metal sites, coordination structures, and atom utilization.	Less emphasis on comparison with broader catalyst families and device-level implementation.	Treats SACs/DACs as one catalyst class and compares their advantages and limitations with nanoparticles, alloys, compounds, and carbon-based composites.
Transition-metal compounds for water splitting	[16,17,18,19]	Summarizes oxides, hydroxides, phosphides, and multimetallic compounds for HER/OER.	Mainly water-splitting oriented, with limited cross-comparison across photocatalysis, CO_2_ reduction, ORR, and hybrid electrolysis.	Links compound catalysts to multiple reactions and highlights how anion chemistry, phase reconstruction, defects, conductivity, and interfaces affect activity and stability.
MOF-derived catalysts and scalable synthesis	[20,21,22]	Highlights porous architectures, precursor designs, and emerging scalable catalyst production.	Synthetic advantages are often separated from reaction descriptors, durability, and industrial operating conditions.	Discusses MOF-derived and related synthesis routes together with scalability, reproducibility, and structural stability concerns.
Photocatalytic water splitting and CO_2_ reduction	[12,23,24,25,26]	Covers light absorption, charge separation, Z-scheme systems, vacancies, cocatalysts, and photocatalytic CO_2_ conversion.	Electrocatalytic descriptors, quantitative benchmarking, and device-level constraints are less central.	Compares photocatalytic and electrocatalytic systems using shared mechanistic logic while distinguishing their different charge-transfer pathways and metrics.
ORR, fuel cells, and metal–air batteries	[27,28,29]	Discusses ORR mechanisms, catalyst classification, alkaline systems, and benchmark discrepancies.	Mostly centered on ORR-related devices rather than broader energy-conversion reactions.	Places ORR within a multi-reaction framework and connects activity descriptors with device-relevant degradation issues.
Industrial translation and sustainability	[21,22,30,31,32]	Emphasizes large-scale production, high-current-density operations, stability, and life-cycle assessments.	Often focuses on selected processes, while catalyst classification and mechanism-guided design are treated as background.	Integrates catalyst design, synthesis, mechanisms, performance comparison, stability, deployment mode, scale-up, TEA/LCA, and life-cycle considerations.
Present review	This work	Covers catalyst families, synthesis strategies, mechanisms, descriptors, enhancement strategies, applications, and translation challenges.	Broad coverage requires cross-comparison rather than exhaustive discussion of every material system.	Provides an integrated roadmap from nanostructure design to practical electro-/photocatalytic energy conversion.

**Table 2:** Classification and design features of nanostructured catalysts for electro- and photocatalytic energy conversion.

Catalyst Family	Defining Feature	Key Design Variables	Main Design Value	Key Concern
Monometallic catalysts	Single-metal nanostructures	Size, morphology, facets, defects, oxidation state	Simple model systems for clarifying structure–activity relationships	Limited electronic tunability; possible aggregation or insufficient activity for complex reactions
Bimetallic/multimetallic alloys	Alloyed, intermetallic, or compositionally integrated multi-metal structures	Composition, atomic ratio, strain, phase, surface segregation	Metal–metal synergy and d-band modulation enable tunable adsorption	Complex active-site identification; possible segregation or reconstruction
Metal compounds	Metal oxides, hydroxides, sulfides, phosphides, nitrides, carbides, and related compounds	Anion chemistry, valence state, vacancies, crystallinity, conductivity	Broad chemical design space with tunable redox and electronic properties	Limited conductivity for some compounds; dynamic surface transformation
Carbon-based composites	Carbon matrices coupled with active phases	Porosity, graphitization, heteroatom doping, dispersion, interface coupling	Improved electron transport, dispersion, and structural stabilization	Active-site ambiguity; possible carbon corrosion or photocorrosion under harsh conditions
Single-atom/dual-atom catalysts	Isolated or paired metal centers on supports	Metal identity, coordination environment, metal–support interaction, dual-site distance	Maximized atom utilization and well-defined sites for mechanistic studies	Aggregation, reconstruction, and uncertain real active configurations

**Table 3:** Comparison of major synthesis strategies for nanostructured catalysts.

Synthesis Strategy	Structural Regulation/Outcomes	Main Advantages	Practical Concerns
Hydrothermal/solvothermal synthesis	Solution-phase crystallization; nanosheets, nanoparticles, hollow, and hierarchical structures	Versatile morphology and facet control	Pressure-limited scale-up; batch uniformity
Electrodeposition/in situ growth	Direct growth on conductive substrates; self-supported films, arrays, and porous coatings	Strong adhesion, low resistance, direct electrode integration	Substrate dependence; large-area uniformity
Template-assisted/MOF-derived synthesis	Confined or precursor-derived structures; hollow, porous, core–shell, and carbon-encapsulated products	Controlled porosity, high surface area, uniform component distribution	Template removal, pyrolysis complexity, possible collapse
Pyrolysis/phosphiding/sulfidation	Thermal phase conversion; phosphides, sulfides, crystalline conductive hybrids	Improved crystallinity and conductivity; tunable anion-regulated electronic structure	Sintering, particle growth, pore loss, atmosphere control
Post-treatment defect/interface engineering	Secondary modification; vacancies, dopants, reconstructed surfaces, interface-rich structures	Fine regulation of active sites, charge transfer, and local electronic structure	Difficult defect quantification; metastable structures

Note: The listed synthesis strategies summarize commonly used approaches for nanostructured catalysts. Structural outcomes, advantages, and practical concerns are general trends based on the literature discussed in Section 3. Specific catalyst examples and references are provided in the main text.

**Table 4:** Key reactions, mechanistic features, and performance descriptors.

Reaction/Process	Core Mechanistic Feature	Key Descriptors	Common Metrics
HER	H* formation and H_2_ desorption	Δ*G*_H*_, charge-transfer kinetics	Overpotential, Tafel slope, exchange current density, stability
OER	Multistep *OH/*O/*OOH oxidation	Oxygen-intermediate adsorption, active-site oxidation state	Overpotential, Tafel slope, Faradaic efficiency, durability
ORR	2e^−^ or 4e^−^ O_2_ reduction pathway	O_2_/*OOH/*O binding, electron-transfer number	Half-wave potential, onset potential, peroxide yield, stability
UOR	Urea adsorption, dehydrogenation, and C–N cleavage	High-valence Ni sites, urea-intermediate adsorption	Onset potential, current density, Tafel slope, stability
CO_2_RR	CO_2_ activation and product-selective proton–electron transfer	*COOH, *CO, *CHO binding; C–C coupling	Faradaic efficiency, product distribution, partial current density, stability
Photocatalytic water splitting	Light absorption, charge separation, and surface redox	Band structure, carrier lifetime, surface reaction kinetics	H_2_/O_2_ evolution rate, AQY, stability
Photocatalytic CO_2_ reduction	Photogenerated electron transfer to adsorbed CO_2_	Band alignment, CO_2_ adsorption, charge lifetime, active sites	Product yield, selectivity, AQY, stability

Note: The descriptors and metrics listed in this table are representative and were summarized based on the literature discussed in Section 4. For CO_2_ reduction, the C–C coupling descriptor specifically applies to multi-carbon product formation.

**Table 5:** Performance enhancement strategies and mechanistic effects of nanostructured catalysts.

Enhancement Strategy	Main Structural Target	Mechanistic Effect	Performance Benefit	Possible Trade-Off	Ref.
Morphology/microstructure control	Size, shape, exposed facets, porosity, hierarchical architecture	Increases accessible active sites and optimizes mass transport	Higher activity and faster reaction kinetics	Complex synthesis; possible structural instability	[114,125]
Elemental doping	Heteroatom or foreign-metal incorporation	Modulates electronic structure, charge distribution, and intermediate adsorption	Improved intrinsic activity and selectivity	Excessive doping may create inactive or unstable sites	[128,129]
Heterostructure/interface engineering	Junctions, phase boundaries, and interfacial contacts	Promotes interfacial charge transfer and synergistic adsorption	Improved charge separation, reaction kinetics, and multifunctional activity	Interface instability or difficult structure control	[106,136,141]
Defect/vacancy engineering	Oxygen vacancies, lattice defects, unsaturated sites	Regulates local coordination, electronic states, and adsorption strength	More active sites and improved surface reaction kinetics	Defects may be metastable under operation	[152,156]
Support/composite modification	Conductive supports, carbon matrices, protective or dispersive phases	Improves dispersion, conductivity, and support–active phase interaction	Better durability, electron transport, and active-site utilization	Weak coupling or support corrosion may limit performance	[167,170]

Note: The listed effects and trade-offs represent general trends. Their actual influence depends on catalyst composition, reaction environment, and operating conditions.

**Table 6:** Application scenarios and practical requirements for nanostructured catalysts.

Application Scenario	Main Catalytic Reactions	Desired Catalyst Properties	Key Metrics	Practical Requirements
Water splitting for H_2_	HER, OER; photocatalytic H_2_/O_2_	Bifunctional activity, charge transfer, corrosion resistance	Overpotential, Tafel slope, H_2_/O_2_ rate, AQY, stability/durability	High-current density, long-term stability, scalable catalyst/electrode fabrication
CO_2_ reduction	Electro- or photocatalytic CO_2_ reduction	Product-selective sites, CO_2_ adsorption, charge transfer	Faradaic efficiency, product distribution, partial current, yield, stability	Suppressed HER, stable selectivity, efficient mass transport, realistic CO_2_ feed
Fuel cells/metal–air	ORR; ORR/OER in rechargeable batteries	ORR/OER activity, conductivity, poisoning resistance, stable active sites	*E*_1_/_2_, onset potential, charge–discharge voltage gap, power density, cycling	Low precious metal loading, air-electrode stability, impurity tolerance, device compatibility
Energy-saving/anodic oxidation	UOR, hydrazine oxidation, alcohol oxidation	Low-potential activity, selectivity, anti-poisoning	Cell voltage, current density, H_2_ rate, product selectivity, stability/durability	Lower energy consumption, feedstock availability, impurity tolerance, value-added product control
Seawater/unconventional electrolysis	Seawater splitting; chloride-resistant OER	Chloride resistance, OER selectivity, corrosion resistance, robust interfaces	Current density, cell voltage, chlorine suppression, durability	Impure-feed tolerance, anti-corrosion design, long-term operation, electrolyte compatibility

Note: The application scenarios, key metrics, and practical requirements are summarized from the literature discussed in Section 6. They represent general trends and typical considerations for nanostructured catalysts; direct comparison across different application scenarios should be made with caution due to variations in reactors, electrolytes, light sources, and device architecture.

**Table 7:** Quantitative comparison of representative nanostructured catalysts in selected electro- and photocatalytic energy-conversion applications.

Application	Catalyst/Structure	Reaction/System	Operating Conditions	Key Quantitative Performance	Stability/Durability	Ref.
Water splitting	NiCoFeP@NiCoFe-LDH medium-entropy heterostructure	Overall water splitting	1 M KOH	Cell voltage: 1.42 V at 10 mA cm^−2^	600 h at 500 mA cm^−2^	[187]
Water splitting	Mn–Co–Fe ternary phosphide nanosheet arrays/Ni foam	Overall water splitting	1 M KOH	Cell voltage: 1.66 V at 100 mA cm^−2^	72 h at 10 mA cm^−2^ in full-cell test	[188]
Photocatalytic water splitting	V-doped Ti-squarate MOF	Photocatalytic overall water splitting	Simulated sunlight, 60 mW cm^−2^; pure water; cocatalyst-free	H_2_ evolution rate: 83 μmol g^−1^ h^−1^ in OWS; simultaneous H_2_/O_2_ evolution	Structural robustness discussed; long-term cycling time not specified	[197]
Photocatalytic H_2_ evolution	Fluorenone-based CTF/twinned Zn_0_._5_Cd_0_._5_S S-scheme heterojunction	Photocatalytic H_2_ evolution	Pt cocatalyst; 0.25 M Na_2_S/Na_2_SO_3_ sacrificial agent	H_2_ evolution rate: 247.62 mmol g^−1^ h^−1^; AQE 42% at 420 nm	No obvious activity decrease after six cycles	[195]
CO_2_ reduction	Ru-doped Ni/ZrO_2_	Photothermal CO_2_ methanation	Photothermal CO_2_ hydrogenation; 170–360 °C high-selectivity range	CH_4_ selectivity: ≈100% over 170–360 °C	Stable phase after reaction; no long-term time specified	[207]
CO_2_ reduction	Ag/Cu_2_O heterojunction	Electrocatalytic CO_2_ reduction to C_2+_ products	Flow cell; 1 M KOH	C_2+_ FE: 77.8% at −300 mA cm^−2^	12 h at −200 mA cm^−2^ with FE(C_2+_) ≈ 70%	[210]
CO_2_ reduction	Cl-doped SnO_2_ nanoflowers/Ni hollow fiber	Electrocatalytic CO_2_ reduction to formate	2 M KHCO_3_; CO_2_ flow: 30 sccm; nickel hollow fiber electrode	Formate selectivity: 99%; CO_2_ single-pass conversion: 93% at 2 A cm^−2^	520 h at 3 A cm^−2^ with formate FE > 94%	[211]
CO_2_ reduction	Cavity-networked Cu nanostructure	Electrocatalytic CO_2_ reduction to C_2_H_4_	pH 1 acidic electrolyte; 0.05 M H_2_SO_4_ + 3 M KCl; MEA configuration	C_2_H_4_ FE: 54.7% at 600 mA cm^−2^; total C_2+_ FE: 71.4%	40 h in MEA configuration	[212]
ORR/metal–air batteries	Fe–N–C with adjacent carbon vacancies	ORR/OER in rechargeable Zn–air battery	Alkaline Zn–air battery	Δ*E* = 0.63 V; peak power density: 218 mW cm^−2^	200 h cycling	[226]
ORR/metal–air batteries	Co single atoms anchored on Co_3_O_4_/N-doped active carbon	ORR/OER in rechargeable Zn–air battery	6 M KOH + 0.2 M Zn(Ac)_2_; rechargeable Zn–air battery	Δ*E* = 0.773 V at 10 mA cm^−2^	35 h cycling at 10 mA cm^−2^	[229]
Energy-saving electrolysis	Phosphorized CoNi_2_S_4_ yolk–shell spheres	Urea electrolysis	1 M KOH + 0.5 M urea	Cell voltage: 1.402 V at 10 mA cm^−2^	100 h	[238]
Energy-saving electrolysis	Ni_2_P_4_O_12_/NiTe heterojunction	Urea electrolysis	Alkaline urea electrolysis	100 mA cm^−2^ at 1.475 V; nearly 100% Faradaic efficiency	>500 h	[239]
Seawater electrolysis	Co_3_S_4_ with tip-enhanced electric field	Seawater electrolysis coupled with sulfion oxidation	Cathode: 1 M NaOH seawater; anode: 1 M Na_2_S + 1 M NaOH	100 mA cm^−2^; chlorine-free H_2_ production; cell voltage reduced by 67.9% vs. conventional seawater electrolysis	HSE stable up to 504 h	[242]
Seawater electrolysis	Fe–Co_2_P/CeO_2_ heterostructure nanosheet arrays	Seawater electrolysis coupled with hydrazine oxidation	1 M KOH seawater + 0.5 M N_2_H_4_	OHzS electrolyzer: 0.014 V at 10 mA cm^−2^ and 0.438 V at 400 mA cm^−2^	10 h at 400 mA cm^−2^	[244]

## Data Availability

No new data were created or analyzed in this study. Data sharing is not applicable to this article.

## Abbreviations

The following abbreviations are used in this manuscript:

AEM	Adsorbate evolution mechanism
AQE	Apparent quantum efficiency
AQY	Apparent quantum yield
CB	Conduction band
CO_2_RR	Carbon dioxide reduction reaction
CTF	Covalent triazine framework
DFT	Density functional theory
EIS	Electrochemical impedance spectroscopy
FE	Faradaic efficiency
HER	Hydrogen evolution reaction
HSE	Hybrid seawater electrolysis
IR	Infrared spectroscopy
LDH	Layered double hydroxide
MEA	Membrane electrode assembly
MOF	Metal–organic framework
M–N–C	Metal–nitrogen–carbon
OER	Oxygen evolution reaction
OHzS	Overall hydrazine splitting
ORR	Oxygen reduction reaction
OWS	Overall water splitting
PL	Photoluminescence
RHE	Reversible hydrogen electrode
SACs	Single-atom catalysts
TEM	Transmission electron microscopy
TRPL	Time-resolved photoluminescence
UOR	Urea oxidation reaction
UV–vis DRS	Ultraviolet–visible diffuse reflectance spectroscopy
VB	Valence band
ZIF	Zeolitic imidazolate framework
